# Cargo-free particles divert neutrophil-platelet aggregates to reduce thromboinflammation

**DOI:** 10.1038/s41467-023-37990-z

**Published:** 2023-04-28

**Authors:** Alison L. Banka, M. Valentina Guevara, Emma R. Brannon, Nhien Q. Nguyen, Shuang Song, Gillian Cady, David J. Pinsky, Kathryn E. Uhrich, Reheman Adili, Michael Holinstat, Omolola Eniola-Adefeso

**Affiliations:** 1 Department of Chemical Engineering, University of Michigan, Ann Arbor, MI 48109 USA; 2 Department of Chemistry, University of California Riverside, Riverside, CA 92521 USA; 3 Division of Cardiovascular Medicine, Samuel and Jean Frankel Cardiovascular Center, University of Michigan, Ann Arbor, MI 48109 USA; 4 Department of Pharmacology, University of Michigan, Ann Arbor, MI 48109 USA; 5 Department of Biomedical Engineering, University of Michigan, Ann Arbor, MI 48109 USA; 6 Macromolecular Science and Engineering Program, University of Michigan, Ann Arbor, MI 48109 USA

**Keywords:** Biomedical engineering, Platelets, Drug delivery, Neutrophils, Drug delivery

## Abstract

The combination of inflammation and thrombosis is a hallmark of many cardiovascular diseases. Under such conditions, platelets are recruited to an area of inflammation by forming platelet-leukocyte aggregates via interaction of PSGL-1 on leukocytes and P-selectin on activated platelets, which can bind to the endothelium. While particulate drug carriers have been utilized to passively redirect leukocytes from areas of inflammation, the downstream impact of these carriers on platelet accumulation in thromboinflammatory conditions has yet to be studied. Here, we explore the ability of polymeric particles to divert platelets away from inflamed blood vessels both in vitro and in vivo. We find that untargeted and targeted micron-sized polymeric particles can successfully reduce platelet adhesion to an inflamed endothelial monolayer in vitro in blood flow systems and in vivo in a lipopolysaccharide-induced, systemic inflammation murine model. Our data represent initial work in developing cargo-free, anti-platelet therapeutics specifically for conditions of thromboinflammation.

## Abstract (web summary)

Platelet-neutrophil aggregates are a hallmark of thromboinflamation. Here, the authors use cargo-free particles to block platelet-neutrophil aggregates’ vascular wall adhesion, which could become an effective thromboinflammation therapy, regardless of disease cause.

## Introduction

Platelets are essential in maintaining hemostasis to prevent excessive blood loss and permanent damage. Their preferential accumulation near the vascular wall in blood flow puts platelets close to the endothelium, a monolayer of endothelial cells (ECs) that line the vessel wall, where they can bind underlying extracellular matrix (ECM) proteins that become exposed when the endothelium is injured or compromised. However, many pathologies are initiated or propagated by platelet interaction with an inflamed or damaged endothelium. In particular, the contribution of platelets to the development of intravascular thrombi (or clots) prominent in many cardiovascular diseases has been well-established, including in ischemic stroke^1^, atherosclerosis^2^, deep vein thrombosis^3,4^, and myocardial infarction^5,6^. A commonality of these diseases is thromboinflammation, or the uncontrolled development of blood clots with inflammation, worsened by an excessive immune response^7^.

Platelets are recruited to an area of inflammation in several ways; platelets may bind to the underlying ECM proteins after endothelium damage or to von Willebrand factor (vWF) multimers or P-selectin released by activated endothelial cells^8^. Once attached, platelets contribute to the inflammatory cascade by releasing alpha- and dense-granules containing adhesion ligands and receptors, such as P-selectin, signaling molecules, including CD40L, CXCL4, and CXCL8, and other inflammatory molecules like histamine and ADP^9,10^. The GPIIb/IIIa receptor activation stabilizes the platelet aggregate by crosslinking vWF or fibrinogen^9–11^. Platelets thus both form and strengthen local thrombi while recruiting other cells to the area of inflammation.

Leukocytes, particularly neutrophils, also contribute to thrombus development upon adhering to an area of inflammation. Neutrophils assist in thrombus development in several ways, including the generation of neutrophil extracellular traps (NETs), which can trap platelets, red cells, and vWF in an area of inflammation^12^, and the release of granular enzymes, including cathepsin G and elastase, that promote the activation of the coagulation system^13^. Additionally, neutrophils and other leukocytes can form aggregates with platelets under thromboinflammatory conditions via binding between PSGL-1 on leukocytes and P-selectin on activated platelets. Circulating neutrophil-platelet aggregates are found in various inflammatory diseases, including sickle cell disease^14^, deep vein thrombosis^15^, and acute coronary syndrome^16^. More recently, circulating platelet-neutrophil aggregates were significantly higher in patients with moderate and severe COVID-19 than in non-COVID controls^17,18^, contributing to the hallmark acute thromboinflammatory phenotype of COVID patients. These neutrophil-platelet aggregates can be recruited to an inflamed or damaged endothelium through interactions between the neutrophil and the endothelium at lower shear rates or binding of platelets to vWF or exposed ECM at higher shear rate^19^.

Many therapeutics have been developed and tested to limit neutrophil-platelet interactions, considering their significant role in the pathogenesis of severe thromboinflammatory conditions. For example, PSGL-1 antagonists were tested in clinical trials in addition to thrombolytic therapies during acute myocardial infarction but did not improve clinical end points^20,21^. Later clinical trials examined the use of an anti-P-selectin antagonist, which did not lead to improved patient outcomes after coronary artery bypass surgery^22^. Other alternatives evaluated in animal models include aspirin-based therapeutics in murine acute lung injury models; though these lessened injury and disease burden^23,24^, the known bleeding risk associated with aspirin may outweigh its potential benefits. Given the importance of platelets and leukocytes in thromboinflammation, many efforts have been made to develop targeted, injectable therapeutics to modulate platelet and leukocyte behavior. However, much of these platelet-targeted therapeutics focus on artificial platelet-like constructs to assist in clotting in situations of trauma or thrombocytopenia, such as fibrinogen-targeted liposomes loaded with tranexamic acid to stabilize clots^25^, liposomes functionalized with collagen, fibrinogen, and vWF binding motifs^26^, and fibrin-binding, ultra-low crosslinked microgels with the ability to contract upon incorporation into a clot^27^. Other therapeutics target platelet-rich clots directly for local delivery of thrombolytics, including Annexin V-targeted, lumbrokinase-loaded micelles^28^.

Alternatively, extensive research has explored targeting drug carriers to a damaged or inflamed endothelium^29,30^ as well as targeting particulate drug carriers to leukocytes, including polymeric particles that reduce the number of infiltrating immune cells in acutely inflamed tissues^31–33^. A prior publication examined the impact of spherical polymeric particles on leukocyte adhesion to an inflamed endothelium in blood flow, where untargeted polystyrene nano- and microparticles decreased leukocyte adhesion to the activated endothelium. Particles conjugated with sialyl Lewis^A^ ligands to bind to E-selectin further reduced cell adhesion^34^. This work suggested that polymeric drug carriers can reduce leukocyte recruitment to areas of inflammation and excessive innate immune cell recruitment. Additionally, particulate drug carriers decreased neutrophil recruitment to the lungs in a murine model of acute lung injury^33^, where neutrophils and neutrophil-platelet aggregates are known to play a damaging role. Despite the promise of model particulate drug carriers in reducing leukocyte adhesion and recruitment in inflammatory models, such drug carriers’ ability to modulate platelet adhesion in thromboinflammation has yet to be studied.

In this work, we evaluated the ability of 200 nm, 500 nm, 2 µm, and 4.5 µm spherical particles, serving as model drug carriers, to modulate platelet adhesion under thromboinflammatory conditions in blood flow in vitro and in vivo. The choice of particle sizes is based on prior literature, where particles in the 100–200 nm size range are of interest for targeted drug delivery applications^35,36^, and particles in the 2–5 µm size range are reported to have superior margination propensity, i.e., ability to localize to the blood vessel wall when compared to nanoparticles^29,37^. We leveraged our recently developed in vitro model of a damaged, inflamed endothelial cell layer that facilitates the adhesion of platelet-leukocyte aggregates in whole blood flows^38^. We demonstrate that micron-sized polymeric particles, serving as model drug carriers, significantly decrease platelet adhesion to inflamed endothelium in vitro by interfering with platelet-neutrophil aggregates’ vascular adhesion via collision and in vivo via a combination of collision and phagocytosis-based platelet-neutrophil aggregate disruption.

## Results

### Polymeric particles modulate leukocyte and platelet adhesion to inflamed endothelium by interfering with leukocyte adhesion

**Fig. 1:**
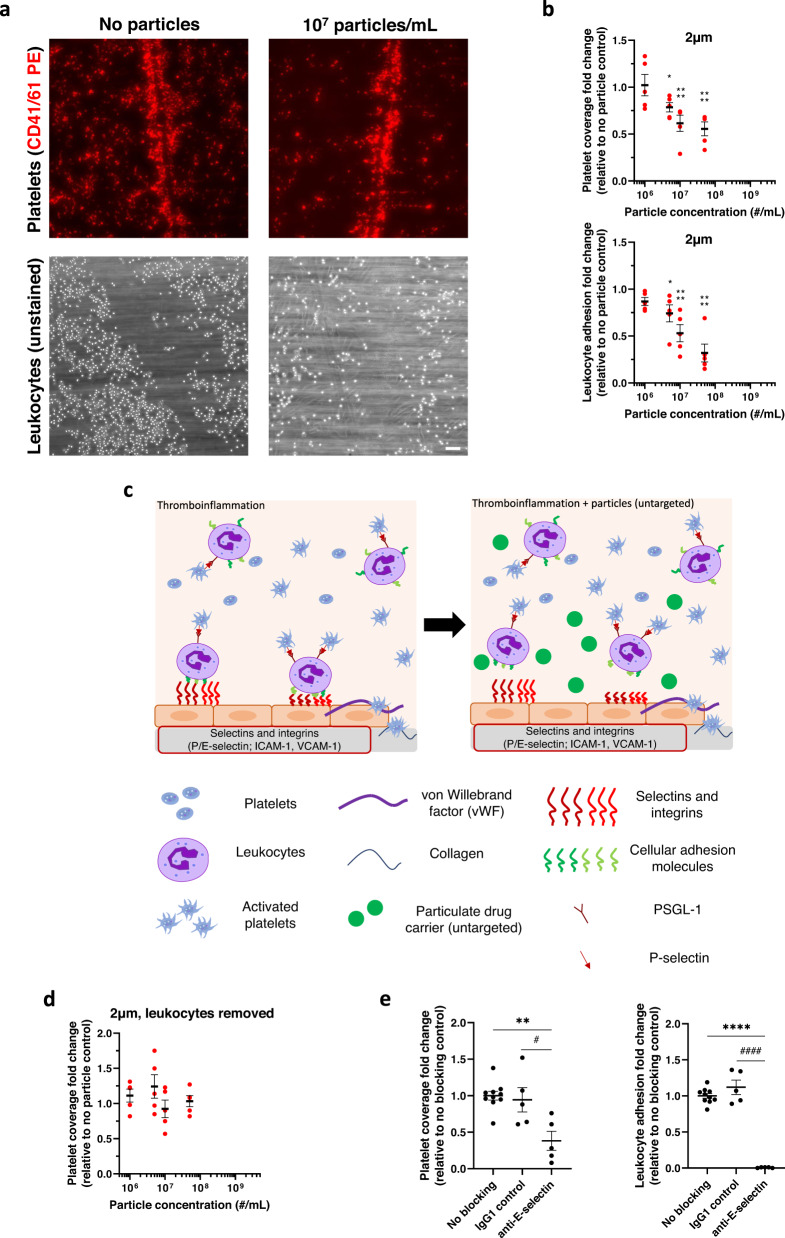
Micron-sized particles decrease platelet-leukocyte aggregate adhesion to an inflamed endothelium. **a** Representative images of adherent platelets (red) and leukocytes (unstained) to inflamed, damaged HUVEC monolayer in the absence (left) or presence (right) of 1 × 10^7^/mL 2 µm IgG-conjugated polystyrene particles. **b** Impact of 2 µm IgG-conjugated model polystyrene particles on platelet adhesion and leukocyte adhesion to an inflamed, damaged endothelium in whole blood flow compared to non-particle controls. **c** Schematic detailing the mechanism of reducing platelet-leukocyte aggregates to inflamed endothelium in vitro due to micron-sized, untargeted polymeric particles. **d** Impact of 2 µm IgG-conjugated polystyrene particles on platelet adhesion to inflamed, damaged endothelium with leukocytes removed from blood, and (**e**) Change in platelet (left) and leukocyte (right) adhesion to endothelium after blocking E-selectin. *N* = 5 independent donors were utilized for each particle treatment (**b**, **d**) or blocking treatment (**e**) with 2 replicates per donor utilized for each (non-treatment) control. Statistical analyses were performed using one-way ANOVA (**d**) with Tukey’s multiple comparisons test or two-way ANOVA (**a**, **c**) with Sidak’s multiple comparison tests. For (**a**) and (**c**), (*) indicates *p* < 0.05, and (****) indicates *p* < 0.0001 in comparison to no particle controls. For (**d**), (**) indicates *p* < 0.01 and (****) indicates *p* < 0.0001 in comparison to no blocking controls and (#) and (####) in comparison to IgG1 controls. Lack of symbols indicates no statistical significance. Circles represent individual data points, horizontal bar represents the average of individual data points, error bars represent standard error, and scale bar = 100 µm. Source data and specific p-values are provided as a Source Data file.

As previously described^38^, our in vitro damaged, inflamed endothelium model supported substantial leukocyte and platelet adhesion when perfused with whole human blood (Fig. 1a). Briefly, we activated a monolayer of HUVECs (endothelium) cultured on a glass coverslip with IL-1β to upregulate expression of cellular adhesion molecules E-selectin and ICAM-1 that support leukocyte adhesion^39^. We further damaged the HUVEC via histamine stimulation and a scalpel score to increase expression of vWF to facilitate platelet adhesion to vWF and the exposed extracellular matrix^38^. We encouraged the formation of platelet-leukocyte aggregates in whole human blood by activating platelets via adenosine diphosphate (ADP), increasing the expression of platelet P-selectin to bind PSGL-1 on the leukocyte surface (Supplementary Fig. 1).

Next, we utilized this blood flow-based damaged endothelium model to elucidate the impact of untargeted, model particulate drug carriers on platelet adhesion under conditions of thromboinflammation. We first explored untargeted particles to determine whether particles without the ability to bind to leukocytes or the endothelium could modulate platelet adhesive behavior based on prior works demonstrating the importance of cell-cell and particle-cell collisions on platelet distribution to the blood vessel wall^40,41^. Representative images of fluorescently stained, adherent platelets and unstained, adherent leukocytes with or without addition of particles are shown in Fig. 1a. Addition of untargeted, 2 µm polystyrene (PS) particles to whole human blood at or above 5 × 10^6^/mL led to a significant decrease in platelet adhesion over the damaged, inflamed endothelium, as measured by the surface coverage of fluorescently labeled platelets (Fig. 1b). Specifically, when polystyrene particles were added to blood at 5 × 10^6^ particles/mL, platelet adhesion dropped to 79% of the adhesion observed for “no particle” controls (*p* = 0.034). Platelet adhesion was further reduced to 62% (*p* < 0.0001) and 53% (*p* < 0.0001) of non-particle controls with higher particle concentrations of 10^7^ and 5 × 10^7^ particles/mL, respectively. When examining the corresponding change in leukocyte adhesion (Fig. 1b), leukocyte adhesion also decreased with increasing particle concentration. However, specifically at high particle concentrations, the fold change in leukocyte adhesion exceeded that of platelet adhesion; for instance, at 5 × 10^7^ particles/mL, leukocyte adhesion decreased to 30% of non-particle controls. (*p* < 0.0001). This finding suggests that particles in blood flow have a more direct effect on leukocyte adhesion to the endothelium than on platelets, which can bind not only to adherent leukocytes but also to vWF strings and the underlying ECM proteins.

Thus, we hypothesized that the mechanism of action for decreased platelet vascular adhesion with particle treatment was due to polystyrene (PS) particles interfering, via collision, with platelet-leukocytes aggregates in freestream, preventing them from adhering to the endothelium as illustrated in Fig. 1c. To interrogate this hypothesis, we removed leukocytes from whole blood by reconstituting platelet-rich plasma with isolated red blood cells. We then repeated flow experiments examining platelet adhesion to an inflamed, damaged endothelium in the presence of untargeted 2 µm PS particles. In contrast to the observation in whole blood (Fig. 1b), there was no change in platelet adhesion with untargeted particles when we removed leukocytes from the system (Fig. 1d), even at high particle concentrations (5 × 10^7^/mL). This finding demonstrated that the impact on platelet adhesion is directly related to the particles’ effect on leukocyte behavior and binding. However, this result did not clarify if leukocytes must adhere to the endothelium for particles to impact platelet adhesion or if the particles’ interaction with leukocytes in freestream is sufficient to have a downstream impact on platelet adhesion.

We next evaluated platelet adhesion in whole blood over an activated (IL-1β) endothelium with leukocyte adhesion blocked via a function-blocking, anti-E-selectin antibody compared to endothelial cells exposed to IgG1 isotype control in the absence of particles. We observed a decrease in platelet adhesion to 38% compared to the non-blocked controls (Fig. 1e, *p* = 0.0015). In contrast, preexposure of the activated endothelium to an IgG1 control did not impact platelet adhesion (*p* = 0.9224). Notably, leukocyte adhesion on E-selectin-blocked endothelial cells decreased to <1% of adhesion on non-blocked controls (*p* < 0.0001) and did not decrease due to a non-blocking IgG1 control (Fig. 1e, *p* = 0.2243). Together with data in Fig. 1d, these results demonstrate that polymeric particles’ interference with leukocyte vascular wall adhesion drives their impact on platelet vascular wall adhesion, i.e., preventing platelet-bound leukocytes from interacting with or remaining adherent to the inflamed endothelial cell surface.

Thus far, non-biodegradable polystyrene polymer particles were used in assays as they are monodispersed and available commercially, allowing exploration of particles’ impact on platelet behavior in thromboinflammation without the complication of size polydispersity. To ensure our findings are consistent regardless of particle material chemistry, we fabricated micron-sized, biodegradable poly(lactic-*co*-glycolic acid) (PLGA) particles (1.83 ± 0.71 µm). PLGA was chosen given its ubiquitous use as base material for drug carriers and is currently utilized in different FDA-approved medical devices^42^. A summary of the physical characteristics of the ~2 µm PS and PLGA particles used in vitro is provided in Supplementary Table 1. The magnitude of PLGA particles’ impact on platelet and leukocyte adhesion at various particle concentrations is shown in Supplementary Fig. 2a, b, with representative images shown in Supplementary Fig. 2c. Supplementary Fig. 3a, b compare the impact of PS and PLGA particles on cell adhesion directly; we observed no significant difference between the reduction in platelet adhesion or the reduction in leukocyte adhesion between 2 µm untargeted PS and PLGA particles, indicating that the observed effects in vitro are dominated by biophysical interactions between cells and particles and not based on polymer material type. Because PS and PLGA behaved similarly in vitro, further experiments utilized PS particles, given their commercial availability as monodispersed samples, unless otherwise stated.

### Micron-sized particles have a more significant impact on platelet adhesion than nano-sized particles

**Fig. 2:**
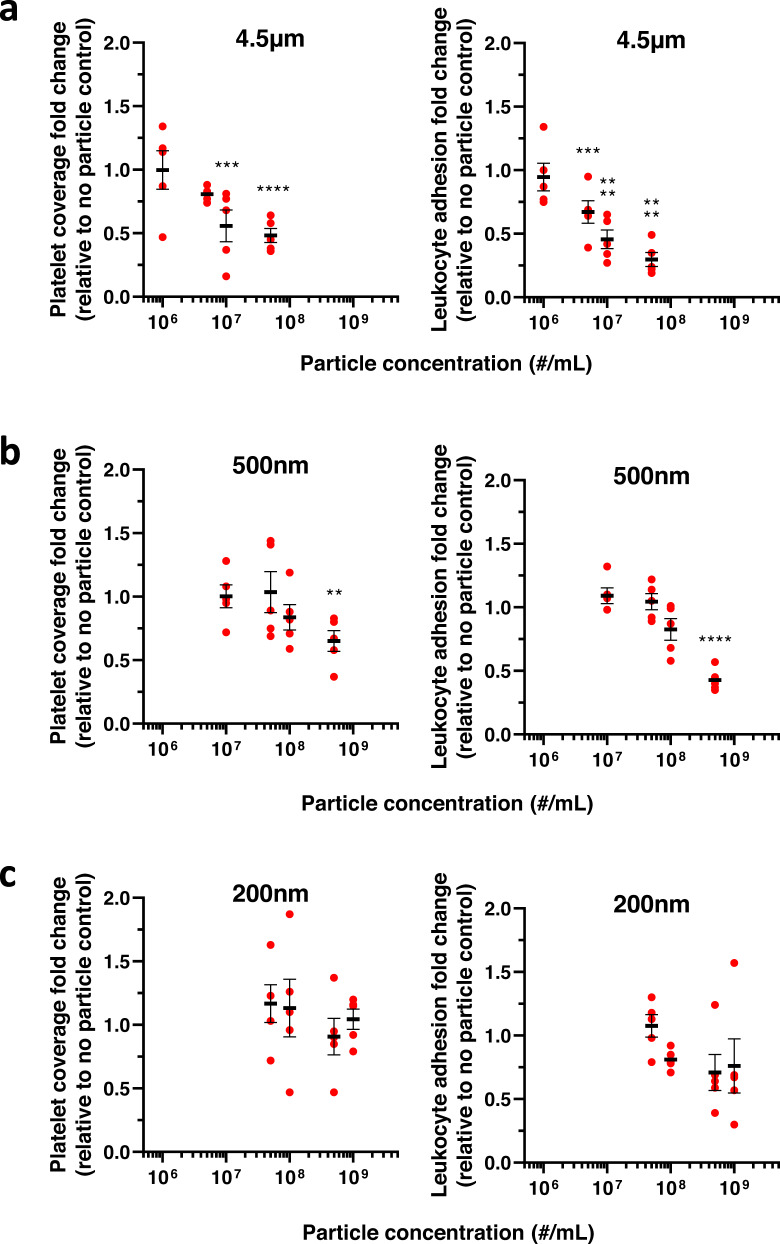
Micron-sized particles outperform nano-sized particles in modulating platelet and leukocyte adhesion to an inflamed endothelium in human blood. Change in platelet and leukocyte adhesion to a damaged, inflamed endothelium with the addition of untargeted (**a**) 4.5 µm, (**b**) 500 nm, or (**c**) 200 nm IgG-conjugated polystyrene particles. *N* = 5 independent donors were utilized for each particle treatment with 2 replicates per donor utilized for each (non-treatment) control. Statistical analyses were performed using two-way ANOVA with Sidak’s multiple comparison tests. (*) indicates *p* < 0.05, (**) indicates *p* < 0.01, (***) indicates *p* < 0.001, and (****) indicates *p* < 0.0001 in comparison to no particle controls. Lack of symbols indicates no statistical significance. Circles represent individual data points, horizontal bar represents the average of individual data points, and error bars represent standard error. Source data and specific p-values are provided as a Source Data file.

Prior publications have reported that particle size matters in their spatial distribution in human blood flows. Micron-sized particles exhibit enhanced margination to the vessel wall, while nano-sized particles primarily localize within the red blood cells (RBC) core^30,43^. Thus, we examined the contribution of three additional particle sizes (200 nm, 500 nm, and 4.5 µm) on platelet and leukocyte adhesion across a range of particle blood concentrations. The 4.5 µm untargeted particles effectively decreased both platelet and leukocyte adhesion at or above particle concentrations of 10^7^, as shown in Fig. 2a. Specifically, at 5 × 10^7^ particles/mL, the 4.5 µm particles decreased platelet adhesion to 47% (Fig. 2a, *p* < 0.0001) and leukocyte adhesion to 24% (Fig. 2a, *p* < 0.0001) of the baseline (i.e., no particle controls). However, this performance did not lead to a more significant reduction in platelet adhesion relative to the impact of the 2 µm particles at the same concentration (Figs. 1b, 2a), suggesting that there is an optimal untargeted particle size for the reduction of platelet adhesion beyond which there is no added benefit to increasing particle size.

Conversely, decreasing particle size to the nanometer range led to a muted impact on platelet and leukocyte adhesion. Untargeted 500 nm particles did not impact platelet adhesion at the maximum concentration evaluated for microparticles (Fig. 2b). Only at a concentration of 5 × 10^8^/mL did the 500 nm particles significantly affect platelet and leukocyte adhesion, which is a 50–100 folds higher particle concentration than the concentrations needed to impact cell adhesion for the untargeted micron-sized particles. At this high concentration, platelet adhesion decreased to 65% (*p* = 0.0013). Leukocyte adhesion decreased to 35% (*p* < 0.0001) of non-particle controls (Fig. 2b). Accordingly, untargeted 200 nm particles did not significantly impact platelet or leukocyte adhesion, even at an ultra-high concentration of 10^9^ particles/mL, which exceeds both the freestream concentration of platelets^44^ and leukocytes^45^ in human blood (Fig. 2c, *p* = 0.9964 and *p* = 0.2151 for platelets and leukocytes, respectively).

### Vascular targeting enhances polymeric particles’ impact on platelet adhesion

**Fig. 3:**
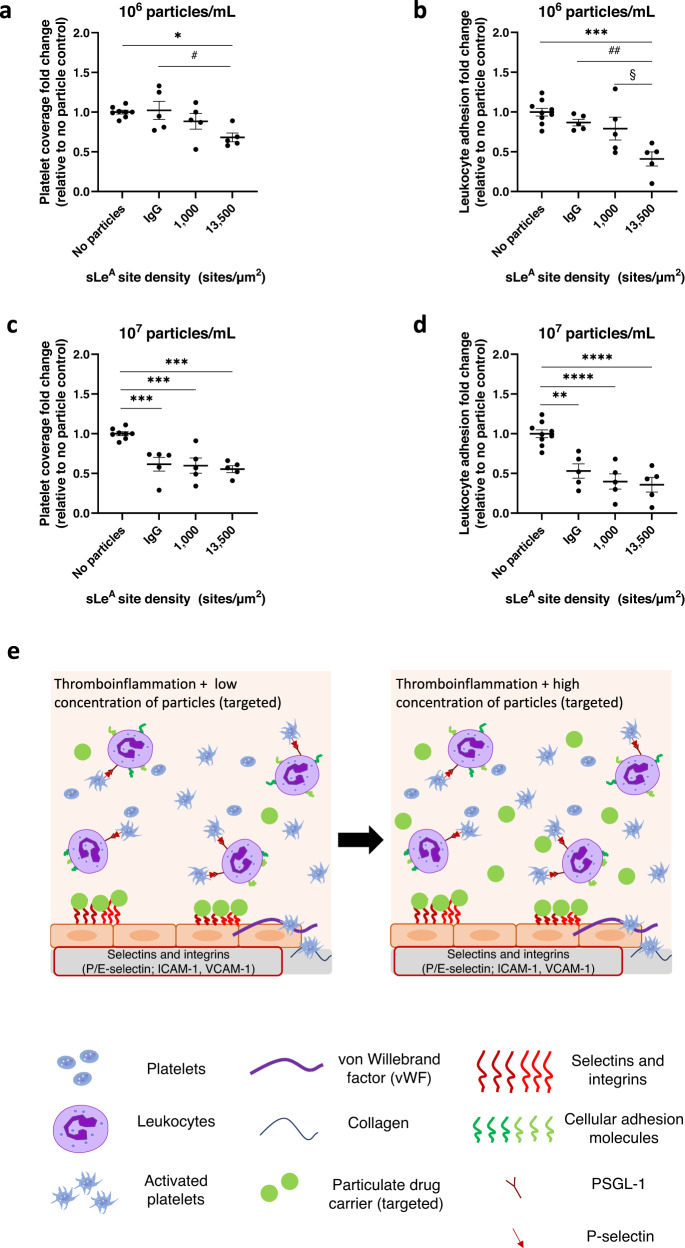
Targeted microparticles outperform non-targeted microparticles at low particle concentration. Impact of 2 µm sialyl Lewis A (sLe^A^)-conjugated particles at 10^6^ particles/mL on (**a**) platelet adhesion or (**b**) leukocyte adhesion. Impact of 2 µm sLe^A^-conjugated particles at 10^7^ particles/mL on (**c**) platelet adhesion, or (**d**) leukocyte adhesion. **e** Schematic of the mechanism of reduction of platelet-leukocyte aggregate binding to an inflamed endothelium in vitro due to the presence of micron-sized, targeted polymeric particles. *N* = 5 independent donors were utilized for each particle treatment (**a**–**d**) with 1–2 replicates per donor utilized for each (non-treatment) control. Statistical analyses were performed using a one-way ANOVA with Tukey’s multiple comparisons. (*) indicates *p* < 0.05, (**) indicates *p* < 0.01, (***) indicates *p* < 0.001, and (****) indicates *p* < 0.0001 in comparison to no particle controls. (##) indicates *p* < 0.01 in comparison to untargeted (IgG conjugated) particles and § indicates *p* < 0.05 in comparison to ‘low’ sLe^A^ targeted particles. Lack of symbols indicates no statistical significance. Circles represent individual data points, horizontal bar represents the average of individual data points, and error bars represent standard error. Source data and specific p-values are provided as a Source Data file.

To further explore the particle design space to determine if we could improve the efficacy of micron-sized particles, especially at low concentrations, we conjugated sialyl Lewis A (sLe^A^) targeting ligands to our 2 µm particle surfaces. sLe^A^ is a small carbohydrate that binds to E-selectin^46^ and can be used as a targeting ligand facilitating particle adhesion to an inflamed endothelium expressing E-selectin^29,34^. We utilized two different sLe^A^ site densities, 1000 sites/um^2^ (‘low’) and 13,500 sites/um^2^ (‘high’), to interrogate the importance of the presence and level of targeting on particles’ ability to impact platelet and leukocyte adhesion. At 10^6^ particles/mL (Fig. 3a), microparticles with high targeting ligand density significantly decreased platelet adhesion to an inflamed endothelium by 32% relative to no particle controls (*p* = 0.019) and untargeted particles (*p* = 0.024). Conversely, particles with low targeting ligand density did not impact platelet adhesion compared to non-targeted particles or the no particles control. The impact of targeted particles on leukocyte adhesion followed a similar trend (Fig. 3b); particles with high levels of targeting yielded a significant reduction in leukocyte adhesion relative to no particle controls (*p* = 0.0002) and untargeted particles (*p* = 0.0076)^34^. No significant reduction in leukocyte adhesion was observed using low sLe^A^ particles. The obtained result is likely due to differences in particle adhesion density to the endothelial cell surface owing to differences in sLe^A^ site density on the particle surface^29^. At the higher particle concentrations of 10^7^/mL tested, the addition of a vascular adhesion ligand did not improve the ability of particles to decrease platelet adhesion over untargeted particles; untargeted particles and targeted particles decreased platelet adhesion to about the same levels (~55–60%) relative to no particle controls (Fig. 3c). Again, the impact of all particle types on platelet adhesion was linked to their ability to decrease leukocyte adhesion (Fig. 3d). Figure 3e illustrates how targeted particles directly compete with leukocytes for binding sites on inflamed endothelial cells, impacting the adhesion of platelet-leukocyte aggregates. At higher particle concentrations, collision impacts likely dominate.

### Micron-sized polymeric particles decrease neutrophil-mediated platelet adhesion to mesentery in a mouse model of systemic inflammation

**Fig. 4:**
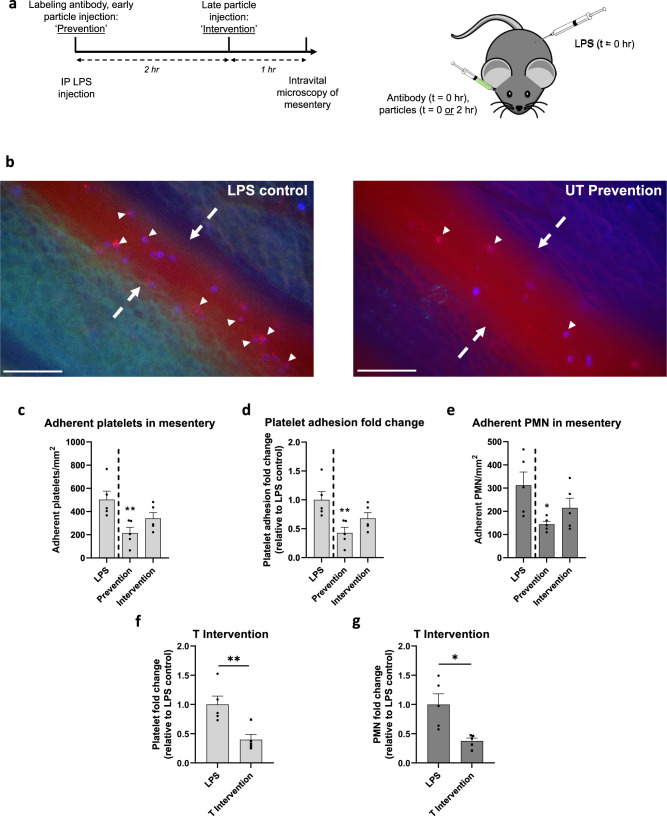
Particles reduce lipopolysaccharide (LPS)-induced neutrophil-platelet aggregate adhesion in mouse mesentery. **a** Timeline, dosing scheme, and experimental schematic of intravital microscopy of inflamed mouse mesentery. Mice received an intraperitoneal (IP) injection of LPS and a retroorbital (RO) injection of labeling antibodies at *t* = 0, and for particle groups, a RO injection of particles at *t* = 0 (‘prevention’) or *t* = 2 h (‘intervention’). Mice were imaged at *t* = 3 h after LPS injection. **b** Representative still image of merged brightfield microscopy, fluorescent Brilliant Violet 421 Ly6G+ neutrophils (blue), FITC polystyrene particles (green), anti-GP1b DyLight 649 platelets (red) channels within mouse mesenteric blood vessel for an LPS-only (non-particle treated; left) and untargeted (UT) Prevention (right) mouse. Neutrophil, platelet, and particle adhesion were quantified only within blood vessels, edges of which are denoted with dashed arrows. Small white arrows highlight several platelet-neutrophil aggregates, scale bar 100 µm. Quantified results of (**c**) platelet adhesion scaled by the surface area of the blood vessel, (**d**) platelet adhesion fold change of UT particle-treated mice relative to LPS-only mice, and (**e**) neutrophil (PMN) adhesion in mouse mesentery 3 h after IP injection of LPS, scaled by the surface area of the blood vessel. Quantified (**f**) platelet adhesion, or (**g**) neutrophil adhesion fold change of T particle-treated mice (intervention) relative to LPS-only mice. Experimental groups consist of *N* = 5 mice per group with 2–4 independent vessels imaged per mouse. Statistical analyses were performed using a one-way ANOVA with Tukey’s multiple comparisons (**c**–**e**) or a two-tailed unpaired Student’s *t* test (**f**, **g**). (*) indicates *p* < 0.05 and (**) indicates *p* < 0.01 in comparison to LPS-only controls (**c**–**e**) or UT intervention (**f**, **g**). Calculated *p*-values are listed when relevant. Lack of symbols indicates no statistical significance. Circles represent individual data points, bar graph represents the average of individual data points, and error bars represent standard error. Source data and specific p-values are provided as a Source Data file.

Next, we utilized a murine model of thromboinflammation to evaluate the effect of microparticles on inflammatory platelet adhesion in vivo to confirm in vitro findings. We aimed to assess the ability of microparticles to reduce platelet adhesion under conditions of acute inflammation. We chose a short-term model due to many diseases, including sepsis, ischemia-reperfusion injury, organ transplant rejection, and COVID-19, characterized by an acute, intense escalation of inflammation and platelet activation and an unmet therapeutic need for blunting this escalation^47,48^. Specifically, we induced endotoxemia in 3–4 weeks old mice (C57BL/6 female) via an intraperitoneal (IP) injection of bacterial lipopolysaccharide (LPS), producing neutrophil-dependent platelet adhesion in the mesenteric vessels^49,50^. A timeline and schematic for this procedure are shown in Fig. 4a; mice were anesthetized and given a retro-orbital (RO) injection of fluorescently conjugated neutrophil and platelet-labeling antibodies with an IP injection of 5 mg/kg LPS. Mice receiving particle treatments fell into two groups: the ‘prevention’ group received 10 mg/kg PS particles at the time of LPS administration. In contrast, the ‘intervention’ group received 10 mg/kg PS particles two hours after LPS administration. Particles were either conjugated with anti-E-selectin and anti-ICAM-1 antibody (‘T,’ targeted) or isotype controls (‘UT,’ untargeted). Mice were anesthetized three hours after being given LPS, and their mesentery examined for adherent neutrophils, platelets, and particles. LPS induces neutrophil and platelet adhesion to the mesentery (Supplementary Movie 1); representative still images of adherent neutrophil-platelet aggregates from an LPS-only control mouse are shown in Fig. 4b (left). Platelets (red) decorate the surface of adherent Ly6G^+^ neutrophils (blue), with some platelet-neutrophil aggregates highlighted with white arrows. Conversely, no neutrophil and minimal platelet adhesion occur in the mesentery for non-LPS inflamed mice (Supplementary Movie 2).

The injection of 2 µm UT PS particles with LPS treatment impacted the adhesion of both platelets and neutrophils to the mesentery wall, as shown in Fig. 4b (right). Specifically, adherent platelets decreased from 503 platelets/mm^2^ for LPS-only mice to 215 platelets/mm^2^ for mice receiving prevention UT particles (Fig. 4c, *p* = 0.0082). Conversely, particle treatment reduced platelet adhesion to 341 platelets/mm^2^ in UT intervention mice. Still, this reduction was not significant (*p* = 0.128) relative to LPS-only mice. Overall, UT particles led to a 57% reduction in platelet adhesion when given at the prevention point (Fig. 4d, *p* = 0.0082). To determine if this decrease in platelet adhesion is accompanied by change in neutrophil adhesion, we also examined the number of Ly6G^+^ neutrophils adherent in the mesentery. Again, only UT prevention particle treatments decreased neutrophils bound to the mesentery wall (Fig. 4e). The number of adherent neutrophils significantly reduced from a maximum of 313/mm^2^ for LPS-only mice to 144/mm^2^ for UT prevention mice (*p* = 0.0252). The neutrophil reduction to 215/mm^2^ for UT intervention was not significant (*p* = 0.205) relative to the no-particle control. Representative movies of UT prevention and UT intervention particle-treated mouse mesentery can be found in Supplementary Movies 3 and 4, respectively. We characterized the fraction of bound neutrophils that are *firmly* adherent versus rolling (i.e., transiently adherent) that passed through the frame throughout each video and found that particle treatments did not significantly change the *percentage* of firmly bound versus rolling neutrophils on the mesentery wall relative to the LPS-only treatment (Supplementary Fig. 4).

Thus far, UT intervention particles, given two hours after installation of LPS, underperformed compared to UT prevention particles given at the same time as LPS. To determine if targeting could improve the efficacy of intervention particles in vivo, as was found with in vitro assays, we added anti-E-selectin and anti-ICAM-1 antibodies to the PS particle surface and examined platelet and neutrophil adhesion to the mesentery wall of these mice. We utilized dual-targeted particles to maximize particle adhesion, as demonstrated by previous researchers^29^. In addition, we replaced sLe^A^ targeting with anti-E-selectin targeting to avoid off-target adhesion of these particles to platelets in this mouse model; sLe^A^ can bind to mouse platelets in vivo via P-selectin on the surface of activated platelets^51^. Raw platelet and neutrophil firm adhesion data for targeted (T) intervention mice are shown in Supplementary Fig. 5. Supplementary Movie 5 represents cell interaction in the mesentery for T intervention particle-treated mice. As shown in Fig. 4f–g, T particles significantly reduced platelet adhesion to 199 platelets/mm^2^ (60% reduction; p = 0.0074) and neutrophil adhesion to 117 neutrophils/mm^2^ (62% reduction; *p* = 0.0107) in comparison to LPS-only mice. The use of T intervention particles thus outperformed UT intervention particles—the latter did not significantly reduce platelet or neutrophil adhesion to the mesentery compared to LPS-only mice (Fig. 4c, d). Interestingly, for all particle injection groups, minimal microparticle adhesion was observed in the inflamed mesentery vessels imaged regardless of the addition of targeting (Supplementary Fig. 6). Due to the utilized in vivo model resulting in systemic inflammation, i.e., throughout the entire mouse, we hypothesize that the adhesion of the dual-targeted particles is dispersed throughout the entire vasculature and thus, not concentrated in the mesentery field of view.

**Fig. 5:**
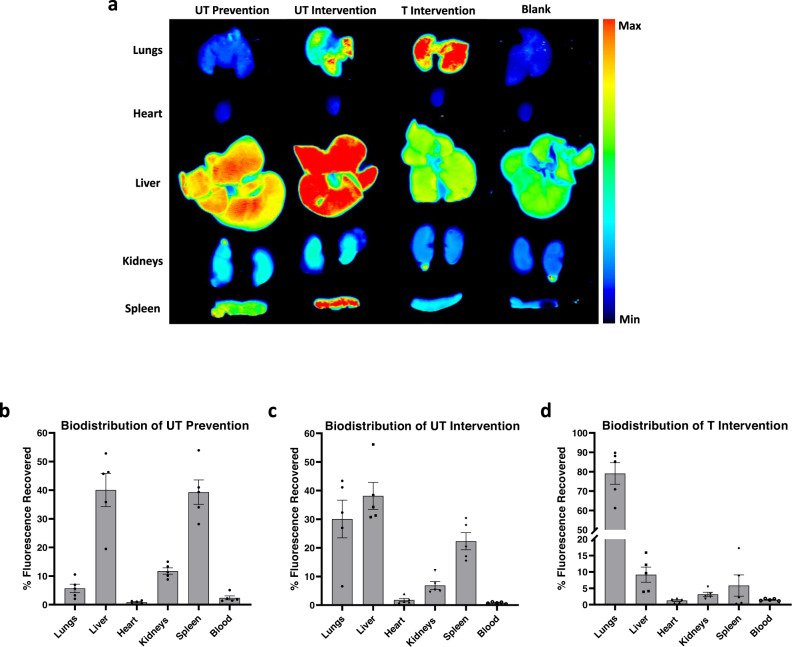
Biodistribution of particles in treated mice. **a** Representative scans of organs treated with particles (‘Untargeted (UT) prevention,’ ‘UT intervention,’ and ‘Targeted (T) intervention’) and untreated (‘Blank’) mice using an Odyssey CLx Infrared Imaging System three hours after treatment with LPS. Quantified results of recovered fluorescence in each organ (percent of total fluorescence recovered from all organs) for mice receiving (**b**) UT prevention, (**c**) UT intervention, and (**d**) T intervention treatments. Experimental groups consist of *N* = 5 mice per group. Circles represent individual data points, bar graph represents the average of individual data points, and error bars represent standard error. Source data are provided as a Source Data file.

To confirm our hypothesis and determine where particles accumulated in vivo, we analyzed the blood and organs three hours after LPS installation for untreated, UT prevention, UT intervention, and T intervention mice. Representative organ scans from each treatment group can be found in Fig. 5a. We find that ~80% fluorescence recovered for the UT prevention mice occurred in the liver and spleen, consistent with natural clearance by filtration and phagocytosis given their non-adhesive nature and the three hours of particle circulation, i.e., particle injection with LPS treatment (Fig. 5b). Accordingly, the fluorescence in UT intervention mice was evenly distributed across the liver, lungs, and spleen (Fig. 5c), aligning with the reduced time particles were in circulation (one hour) at the time of imaging, i.e., particle injection 2 h after LPS. Conversely, ~80% of the fluorescence recovered in T intervention mice was found in the lungs (Fig. 5d), indicating enhanced lung retention over UT intervention at the same time, consistent with targeted particles adhering in the inflamed vasculature of the lungs due to systemic LPS installation.

**Fig. 6:**
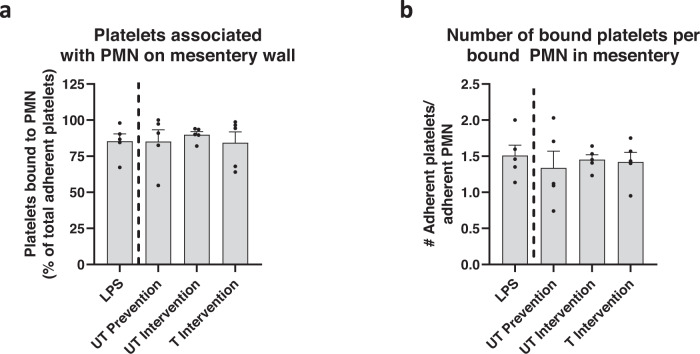
Polystyrene (PS) particle treatment does not alter phenotype of neutrophils in inflamed mouse mesentery. **a** The percent of total platelets adherent to mesentery that are associated with a bound neutrophil (PMN) (of total number of platelets bound to vascular wall). **b** The average number of platelets adherent to each adherent neutrophil in the mesentery. Experimental groups consist of *N* = 5 mice per group with 2–4 independent vessels imaged per mouse. ‘LPS’ refers to lipopolysaccharide, ‘UT’ to untargeted, and ‘T’ to targeted particles. Statistical analyses were performed using a one-way ANOVA with Tukey’s multiple comparisons. Lack of symbols indicates no statistical significance. Circles represent individual data points, bar graph represents the average of individual data points, and error bars represent standard error. Source data and specific *p*-values are provided as a Source Data file.

We next examined the percentage of platelets associated with neutrophils at the vascular wall to ascertain whether the impact of particles on platelet adhesion in vivo was due to particles’ direct impact on platelet adhesion or decrease in platelet-bound neutrophils at the mesentery wall indirectly impacting platelet adhesion. We found that 85% of the platelets bound to the wall in the LPS-only treatment were directly attached to a firmly adherent neutrophil, in contrast to binding to the vascular wall; this percentage did not change with particle treatment (Fig. 6a), suggesting that particle impact on platelets is through impact on neutrophil adhesion similar to in vitro observation. To confirm that particles’ effect on platelet adhesion is due directly to particles removing or blocking neutrophils from the mesenteric wall, we depleted neutrophils from two groups of mice before LPS installation using an anti-Ly6G depletion antibody; one group of mice also received a UT prevention treatment of PS particles. After three hours, both groups of neutrophil depletion mice significantly reduced the number of adherent platelets in the mesentery (Supplementary Fig. 7) compared to LPS-only controls, i.e., with no neutrophil depletion. The UT prevention particle treatment did not further reduce platelet adhesion in neutrophil-depleted mice. Representative movies of neutrophil-depleted mice and neutrophil-depleted mice with UT prevention particles can be found in Supplementary Movies 6 and 7, respectively.

Next, we quantified the average number of platelets bound to neutrophils per *firmly* adherent neutrophil; for all experimental groups, on average, each firmly attached neutrophil was associated with >1 adherent platelet (Fig. 6b). Because each bound neutrophil was associated with at least one bound platelet and at least 84% of all adherent platelets were attached directly to neutrophils (Fig. 6a), even a modest decrease in adherent neutrophils due to particle treatment had a sizeable downstream impact on platelet adhesion, diverting platelets away from an area of inflammation.

Our in vitro results (Fig. 2) highlighted that micron-sized particles were better able to divert platelets from an inflamed endothelium than nano-sized particles. However, to determine if this finding is translatable in vivo, we utilized UT prevention 500 nm particles in the same LPS-induced systemic inflammation murine model. Unlike their micron-sized counterparts, UT prevention nanoparticles did not significantly reduce platelet or neutrophil adhesion compared to LPS controls (Supplementary Fig. 8a, b). A representative movie of a UT prevention nanoparticle-treated mouse mesentery can be found in Supplementary Movie 8.

### Biodegradable, therapeutic polymer microparticles reduce platelet and neutrophil adhesion to mouse mesentery and alter neutrophil phenotype

Next, we evaluated whether use of microparticles constructed from a salicylate-based poly(anhydride-ester) polymer, termed Poly-Aspirin (“Poly-A”), will confer an added anti-inflammatory effect in our in vivo thromboinflammation model. The Poly-A particles are of interest here, given our recent work demonstrating that salicylate-based particles prevented neutrophil migration into airways in a mouse model of acute respiratory distress (ARDS); these Poly-A particles further reduced neutrophil migration compared to PS or PLGA particles via additional anti-inflammatory effects conferred by salicylate in the particle’s polymer backbone^32^. Thus, we hypothesized that Poly-A particles would grant added therapeutic benefit in reducing neutrophil-platelet aggregation and block neutrophil adhesion in vivo in our murine model of thromboinflammation. Particles in the following experiments were unconjugated (no surface modification) as control experiments demonstrated that unconjugated and untargeted but coated with IgG (i.e., UT) PS particles yielded the same impact on platelet and neutrophil accumulation in the mouse mesentery (Supplementary Fig. 9); physical characteristics of the fabricated Poly-A particles are provided in Supplementary Table 1. The Poly-A particles were evaluated in the intervention scheme where UT PS particles did not significantly impact platelet adhesion (see Fig. 4c-d), making any added Poly-A benefits easy to visualize.

**Fig. 7:**
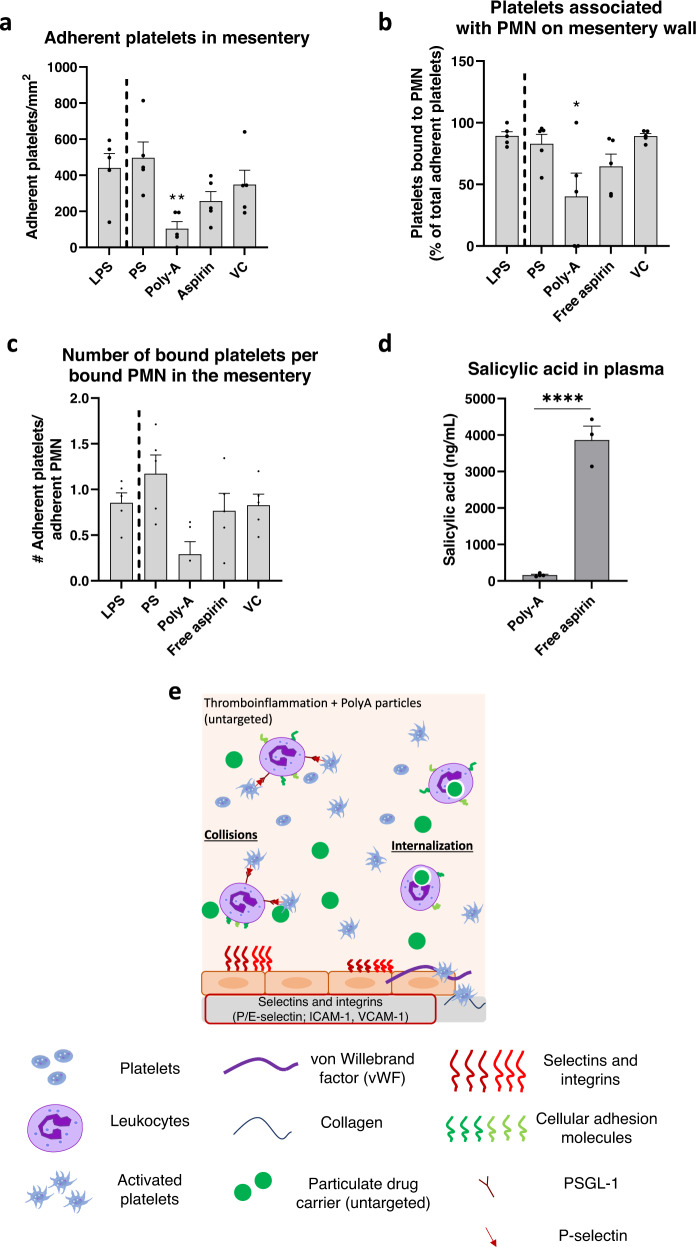
Degradable Poly-A microparticle treatment provides additional anti-inflammatory impact beyond polystyrene (PS) particles in inflamed mouse mesentery. **a** Platelet adhesion scaled by the surface area of the blood vessel. **b** The percentage of total platelets on the mesentery associated with a bound neutrophil (PMN). **c** The average number of platelets adherent to each adherent neutrophil in the mesentery. **d** Salicylic acid present in mouse plasma at the time of euthanasia as measured by liquid chromatography with tandem mass spectrometry (LC-MS/MS). **e** Schematic detailing the mechanism of reducing platelet-leukocyte aggregates to an inflamed mouse mesentery in vivo due to micron-sized, Poly-A polymeric particles. Experimental groups consist of *N* = 5 mice per group with 2–4 independent vessels imaged per mouse and *N* ≥ 3 for salicylic acid measurements. Statistical analyses were performed using a one-way ANOVA with Tukey’s multiple comparisons (**a**–**c**) or a two-tailed unpaired Student’s *t* test (**d**). (*) indicates *p* < 0.05 and (**) indicates *p* < 0.01 in comparison to LPS-only controls. (****) indicates *p* < 0.0001 compared to mice receiving Poly-A particles (**d**). Lack of symbols indicates no statistical significance. Circles represent individual data points, bar graph represents the average of individual data points, and error bars represent standard error. ‘VC’ = vehicle control (10% DMSO in PBS) for the free aspirin assay. ‘LPS’ = lipopolysaccharide. Source data and specific p-values are provided as a Source Data file.

We observed that unconjugated (UC) Poly-A microparticles significantly decreased the number of platelets bound to the vascular wall relative to LPS-only mice when given as an ‘intervention’ treatment (Fig. 7a, *p* = 0.0099). In contrast, UC PS particles did not have a significant effect, similar to UT PS particles (Fig. 4). Notably, Poly-A particles significantly reduced the percentage of adherent platelets associated with firmly bound neutrophils on the mesentery wall to 40% of the total number of platelets adherent in the vessel (Fig. 7b, *p* = 0.0106) and reduced the average number of platelets bound to each firmly adherent neutrophil on the vessel (Fig. 7c, *p* = 0.0647); none of the PS particle groups, regardless of conjugation, targeting, or injection time, impacted any of these characteristics (see Fig. 6). To confirm that the apparent added benefit of Poly-A particles was linked to the microparticle form rather than free salicylic acid released from the particles’ degradation in vivo, we evaluated the impact of systemic aspirin on cell adhesion (‘free aspirin,’ Fig. 7). Notably, treatment with free aspirin did not impact platelet adhesion to the mesentery, nor the percentage of platelets associated with bound neutrophils (Fig. 7). Importantly, measurement of blood one hour after treatment, i.e., at the time of imaging, showed minimal free salicylic acid in mice treated with Poly-A compared to those treated with aspirin (Fig. 7d; *p* < 0.0001). This measurement highlights the essential role of particulate salicylic acid in diverting inflammatory cells from an area of inflammation, likely due to the phagocytosis of Poly-A particles, as previously reported^32,33^. A schematic of how untargeted Poly-A particles impact the adhesion of platelet-leukocyte aggregates to an inflamed mouse mesentery is shown in Fig. 7e. A representative movie of UC Poly-A particle-treated mice can be found in Supplementary Movie 9. To ensure that the salicylic acid-based Poly-A particles had no off-target effects on immune cells or liver function, we evaluated the health of mice receiving daily doses of Poly-A particles. After five days of particle injections, healthy mice receiving Poly-A had no change in the distribution of circulating leukocytes and showed no signs of liver toxicity as measured by an aspartate aminotransferase (AST) activity assay (Supplementary Fig. 10).

## Discussion

There is great interest in injectable drug carrier-based therapeutics to modulate platelet behavior, either to improve clotting in trauma or thrombocytopenia^25,27,52^ or to lyse excessive clots in conditions of thrombosis^28,53^. To our knowledge, this work uniquely demonstrates, in vitro and in vivo, that cargo-free microparticles can redirect platelets away from an area of inflammation via alteration in leukocyte adhesion. In vitro, we established that untargeted micron-sized polymeric particles reduce platelet adhesion to an inflamed, damaged HUVEC monolayer by interfering with platelet-leukocyte aggregates bound to the endothelium. This anti-platelet impact of microparticles was neutralized entirely by removing leukocytes from whole blood. We similarly observed a reduced platelet adhesion by blocking the interactions between leukocytes on activated/inflamed HUVEC monolayer using an anti-E-selectin blocking antibody. When exploring the particulate carrier design space, it is unsurprising that micron-sized particles had a more significant impact than nano-sized particles on cell adhesion in blood flow. While nano-sized particulate drug carriers are often utilized due to their long circulation times in vivo^35^ and for leveraging the enhanced permeability and retention effect in cancer^36^, extensive prior work demonstrates that nano-sized carriers do not effectively localize to the vascular wall, particularly in human blood flow^30,43^. Previous work also showed that nano-sized PS particles with or without active sLe^A^ targeting have a muted ability to interfere with leukocyte adhesion to an inflamed HUVEC monolayer compared to micron-sized particles^34^. Our results thus align with this previous research and demonstrate that micron-sized particles outperform nano-sized particles at decreasing platelet adhesion in conditions of thromboinflammation due to their superior ability to reach the vascular wall.

While untargeted (IgG-coated) particles utilized with in vitro assays did not bind to the inflamed HUVEC monolayer to compete with leukocytes for binding sites, directly targeting particles to a specific injured area may be beneficial in vivo as opposed to using untargeted particles. For instance, platelet-leukocyte aggregates bind in the lungs during acute lung injury and contribute to excessive, localized inflammation^24^. Indeed, adding a vascular adhesive feature to PS particles enhanced their interference with platelet adhesion, specifically at low particle concentration (10^6^/mL), where untargeted PS particles had little-to-no impact on cell adhesion. We utilized ACD anticoagulant for all experiments in vitro, which chelates calcium and prevents or considerably slows calcium-mediated processes. Because phagocytosis is a calcium-mediated process, particles impact the adhesion of platelet-leukocyte aggregates mainly via near-wall collisions removing or interfering with leukocytes bound to the endothelium in vitro. Vascular adhesive particles that bind to the IL-1β-inflamed HUVEC exaggerated the particle-blocking impact via direct competition with leukocytes for binding sites on the endothelium. Our data also suggest a specific particle concentration threshold where the addition of targeting does not further improve particle efficacy, likely because particles have already saturated the system. This observation aligns with previous in vitro work where increasing particle concentration in blood resulted in a large amount of particle localization in the red blood cell-free layer near the vascular wall; hence, particle-cell collisions overwhelm any benefit of particle adhesion at sufficiently high particle concentrations^34,43^.

We confirmed our in vitro findings in vivo in a murine model of systemic inflammation using LPS, using real-time, in vivo imaging to examine neutrophil-dependent platelet adhesion to the mesentery^49,50^. The PS microparticles, both untargeted and targeted to E-selectin and ICAM-1, reduced platelet and neutrophil adhesion to the mesentery. Conversely, nano-sized PS particles did not reduce platelet or leukocyte adhesion in the mesentery. This indicates that our in vitro data showing micron-sized particles outperforming nano-sized particles is translatable in vivo. The addition of targeting antibodies to the particle surface enhanced their anti-thromboinflammatory activity, demonstrating the ability to reduce adherent platelets when injected into blood well after inflammation initiation (~2 h), though without significant adhesion of the particles themselves to the mesentery. We speculate that, due to systemic inflammation caused by an IP injection of LPS, particles may be adhering throughout the mouse vasculature, decreasing their concentration in our field of view. In particular, this model of systemic inflammation is known to induce inflammation and recruit neutrophils to many organs/tissue such as the brain^54^, liver^55,56^, lungs^55–57^, and the peritoneum^57^. Our biodistribution experiments confirmed this hypothesis as the majority of fluorescence for mice receiving targeted particles occurred within the lungs, which are known to have enhanced ICAM-1 expression with systemic inflammation^58,59^, accounting for the low number of adherent particles in the mesentery. Given the retroorbital injection route, the particles will pass through the lungs first before the mesentery bed, explaining the high lung particle trapping there^60^. Conversely, most recovered fluorescence in mice receiving untargeted particles occurred within the liver and spleen, suggesting that untargeted particles were being cleared from circulation. By depleting mouse neutrophils before LPS installation, we confirmed that the reduction of platelets in the mesentery due to PS microparticles depends on neutrophils, like our in vitro results.

Like in vitro assays, we hypothesize that polymeric PS microparticles remove adherent neutrophils from the mouse mesentery through a combination of near-wall collisions and neutrophils phagocytosis of these particles. Indeed, previous work utilizing human neutrophils demonstrated that phagocytosis of PS particles altered the cell surface’s expression of cellular adhesion molecules, ultimately leading to an increase in rolling velocity over an inflamed endothelium in vitro^34^. Because particle phagocytosis can reprogram^32^ and reroute immune cells from areas of inflammation^31–33,61^, we expect that injection of particles diverts neutrophils from the mesentery and has a downstream impact on adherent platelets. Further, we speculate the differences in effects in vivo between PS and Poly-A in the ‘intervention’ assay are due to added therapeutic effect of salicylic acid on neutrophil activation, as described in a recent publication^32^. Specifically, neutrophils that phagocytosed Poly-A particles in vitro in human blood and in vivo in mice demonstrated significant changes in the surface expression of inflammatory molecules, e.g., L-selectin, relative to cells with no particles or cells that phagocytosed PS or PLGA^32^. This change in neutrophil activation may lead to weaker interactions with platelets, as demonstrated by the unique, significant reduction in the number of platelets bound to each neutrophil with Poly-A microparticle treatment.

Overall, these results show that cargo-free polymeric particles decrease acute platelet adhesion to an activated HUVEC monolayer in vitro and the mesentery vascular wall in vivo mainly by interfering with the firm adhesion of platelet-leukocyte aggregates to the endothelium. This finding is critical given the prominence of platelet-leukocyte aggregates in several inflammatory ailments, i.e., thromboinflammation. Indeed, the presence of platelet-leukocyte aggregates indicates severity in many acute and chronic inflammatory diseases, including chronic cardiovascular diseases^16,62–64^, COVID-19^17,18^, and sepsis^65^; and are thus a target for treatment. However, the bulk of previously explored therapies for thromboinflammation has focused on other aspects of pathophysiology, such as anticoagulants to combat the dysregulated coagulation cascade^47,66^, aspirin, and other anti-platelet compounds to directly modulate the reactivity of platelets^67,68^, or focus on injectable therapeutics to platelets, either for imaging^69^, as platelet mimetics to assist in clotting^25–27,52^ or to deliver localized thrombolytic therapies to reduce the burden of dangerous thrombi without increased bleeding risk systemically^28,53^. Only recently have treatments turned attention to the leukocyte component of thromboinflammation. For example, Li et al. demonstrated that AKT2 inhibition reduced neutrophil adhesion and neutrophil-platelet aggregates in a mouse model of sickle cell disease^70^. Others have explored blocking leukocyte adhesion molecules, such as P-selectin^22,71,72^. The blocking of IL-1 receptor, L-selectin, or ICAM-1 has been shown to reduce ischemic injury via reduction of neutrophil-platelet aggregates in preclinical stroke models^73^. However, these approaches have had limited success in clinical trials—e.g., Enlimomab, an anti-ICAM-1 antibody, worsened outcomes in stroke patients^74,75^. The difficulty of targeting a singular soluble factor or adhesion receptor likely lies in the complexity and redundancy inbuilt into the inflammatory response cascade—for every molecule taken out of commission for neutrophil recruitment, either there is a replacement that can perform a similar role, or the molecule plays such an innate physiological role that a blockade can cause adverse side effects that include suppression of the immune system and development of cancer^76^. Unfortunately, unlike in preclinical animal models, no unique cell surface marker allows rapid and transient depletion of neutrophils; therein lies the benefit of a particle-based approach to neutrophil blocking in thromboinflammation. Thus, extensive work has explored using polymeric particles and other drug carriers to reroute immune cells from localized injury or inflammation^31–33,61,77,78^.

Here, we extended the work, demonstrating polymeric particles can divert platelets from an area of inflammation by interfering with the adhesion of platelet-leukocyte aggregates. This direct engagement of critical immune cells is beneficial because it can be effective irrespective of the underlying cause of thromboinflammation. Importantly the presented results suggest the immune-blocking effect conferred by particles occurs regardless of the polymeric material. An enhanced understanding of the physical particle parameters to target a specific leukocyte subset can ensure limited off-target effects—rod-shaped particles appear promising for targeting neutrophils in this regard^79^. Adding a therapeutic component, as in the case of Poly-A microparticles, can enhance treatment potency and should be further explored in additional chronic thromboinflammatory models. As platelet-leukocyte aggregates play a significant role in different diseases^9,80–83^, this work represents a vital step in developing thromboinflammatory therapeutics.

## Methods

### Study approvals

Informed, written consent was obtained by all human blood donors before blood draws according to a protocol (#HUM00013973) approved by the University of Michigan Internal Review Board (IRB-MED). Both male and female donors, aged 18–49, were included, and sex was not considered or found to be a biological variable, given the biomechanical focus of the particle interactions in blood. All participants were monetarily compensated for their blood donation. Under an IRB-MED-approved human transfer protocol (#HUM00026898), umbilical cords (Mott Children’s Hospital, Ann Arbor) were acquired. All animal studies were conducted following the National Institute of Health guidelines for the care and use of laboratory animals and protocol (#PRO00010572) approved by the University of Michigan Institutional Animal Care and Use Committee (IACUC). C57BL/6 female mice 3-4 weeks old were purchased from Jackson Laboratories and maintained in pathogen-free facilities at the University of Michigan.

### Production of poly(lactic-co-glycolic acid) (PLGA) microparticles

Poly(lactic-*co*-glycolic acid) (PLGA) micro-particles were fabricated following an emulsion solvent evaporation technique. Briefly, 30 mg of PLGA polymer (24–38 kDa; Sigma Aldrich) were dissolved in 20 mL of dichloromethane (oil phase). The oil phase was emulsified into a 90 mL PVA/poly(ethylene-alt-maleic anhydride) (PEMA) mixture (81 mL of 1% PVA and 9 mL 2% PEMA; aqueous phase) using an overhead stirrer. Rhodamine B (~0.05 mg per mg polymer) was included in the oil phase to produce fluorescent particles. The emulsion was stirred continuously at 2500 rpm for two hours, allowing for solvent evaporation. The resultant PLGA particles were washed with deionized water via centrifugation. Unincorporated Rhodamine B was removed from the particles during these wash steps. PLGA particles were lyophilized and stored at −20°C until further use. The particles were imaged using scanning electron microscopy (University of Michigan EMAL). Images were analyzed using ImageJ to calculate the average particle size; >50 representative particles were measured to calculate the average particle size.

### Production of poly(anhydride-ester) or Poly-Aspirin (Poly-A) microparticles

Non-fluorescent Poly-A microparticles were fabricated via an emulsion solvent evaporation process. The oil phase consisted of 40 mg of Poly-A polymer^84^ (8–10 kDa, adipic acid linker) dissolved in 2 mL of dichloromethane, and the water phase was 4 mL of 1% PEMA. The oil and water phases were cooled on ice for 10 min before emulsification to prevent polymer damage. Later, the oil and water phases were emulsified at 40% amplitude via sonication (Fisher Scientific Model 50 Sonic Dismembrator) for 2 min. The particle solution was then stirred in 10 mL of 0.5% PEMA for 2 h for solvent evaporation. The resultant Poly-A particles were washed with deionized water via centrifugation and filtered using 2 µm filter tips (Agilent Technologies). Poly-A particles were lyophilized and stored at −20°C until further use. Particles were imaged using scanning electron microscopy and particle size was determined using ImageJ; >50 representative particles were measured to calculate the average particle size.

Fluorescent Poly-A particles were fabricated similarly to non-fluorescent Poly-A particles, except for the oil phase containing 39.6 mg of unlabeled Poly-A polymer and 0.4 mg of Cy5.5-labeled Poly-A polymer. Fabrication of Cy5.5-labeled PolyA-polymer followed ECD/NHS chemistry as previously described^32^.

### Estimation of salicylic acid released from Poly-A microparticles

Unlabeled Poly-A microparticles were dissolved in 1 M NaOH overnight to allow for complete particle degradation. The degradation media was analyzed via ultraviolet-visible spectroscopy and absorbance measurements were obtained at λ = 298 nm. Estimation of total salicylic acid released from the microparticles was calculated against a calibration curve of known concentrations. All salicylic acid standards were also prepared in a solution of 1 M NaOH.

### Functionalization of particles

Carboxylated PS particles (200 nm, 500 nm, 2 µm, 4.5 µm; Polysciences), were conjugated with NeutrAvidin (Thermo Fisher Scientific) through carbodiimide chemistry^30^. In untargeted in vitro flow experiments, the avidin-functionalized particles were then conjugated with biotin rat IgG2b (Biolegend #400604) to prevent non-specific binding to the endothelium. To quantify IgG2 site density, particles were stained with 12 µg/mL anti-rat IgG2b-phycoerythrin (PE, Biolegend #408214). The stained particles and standard beads with known fluorescence (MESF beads, Bangs Laboratories) were analyzed using flow cytometry. The MESF beads generated a calibration curve between fluorescent intensity and the number of fluorescent molecules on the particle. IgG2 site densities were held constant at 1000 sites/µm^2^. The concentration of biotin rat IgG2b required for this site density varied from ~0.01 to 5.00 µg/mL depending on particle size, so IgG2b site density was confirmed daily. For targeted experiments, avidin-functionalized particles were conjugated with biotinylated sialyl Lewis A (sLe^A^), stained with 12 µg/mL anti-CLA-PE (Miltenyi Biotec #130-091-635), and site density quantified. sLe^A^ site densities were held at 1000 sites/µm^2^ for the ‘low’ site density targeting experiments and 13,500 sites/µm^2^ for the ‘high’ site density targeting experiments; the concentration of sLe^A^ varied from ~0.1 to 1.0 µg/mL depending on the desired site density, which was confirmed daily.

For in vivo intravital experiments, avidin-conjugated 2 µm or 500 nm PS particles were conjugated with both anti-E-selectin (R&D Systems #BAM5752) and anti-ICAM-1 (Biolegend #116103) for targeted experiments or anti-IgG2b isotype controls for untargeted experiments. The site density for dual-targeted particles was 50,000 sites/µm^2^ total, with 30,000 anti-E-selectin sites/µm^2^ (~1–3 µg/mL required for conjugation) and 20,000 anti-ICAM-1 site/µm^2^ (~1–3 µg/mL required for conjugation). The site density for untargeted particles was constant at 30,000 anti-IgG2b sites/µm^2^ (~0.4–1.5 µg/mL required for conjugation); site density was quantified as described for the carboxylated PS particles used in in vitro assay and prior publications, and confirmed daily before use^29,30^.

PLGA microparticles were also conjugated with avidin for two hours the night before use using carbodiimide chemistry. To prevent particle degradation, particles were centrifuged, and all liquid was removed before storing the particles at −20 °C. The following day, particles were conjugated with 0.5–1.0 µg/mL anti-IgG2b (1000 sites/µm^2^), and their site density was calculated daily before use. The conjugated particles were used the same day for in vitro experiments.

### Blood draw and preparation for flow experiments

Human whole blood was acquired via venipuncture and immediately anticoagulated with acid citrate dextrose (ACD). Blood was held at 37 °C until use. For select experiments, leukocytes were depleted from whole blood. First, whole blood was spun at 200 g for 15 min to remove platelet-rich plasma. Phosphate-buffered saline (PBS) and dextran were added to the remaining blood, and the RBCs were allowed to settle for 2 h. The remaining plasma and leukocytes were discarded, and the RBCs were washed twice in PBS. Platelet-rich plasma and RBCs were combined at the original hematocrit of each donor before use in flow experiments.

### Preparation of endothelial monolayer

Human umbilical vein endothelial cells (HUVEC) were isolated from acquired umbilical cords using a collagenase perfusion technique. HUVEC were grown in T75 flasks at 37 °C and 5% CO_2_ until confluent and then seeded at a confluent density onto glutaraldehyde-crosslinked gelatin coated 30 mm glass round coverslips (Warner Instruments). Coverslips were utilized 36–48 h after seeding.

HUVEC were activated with 1 ng/mL IL-1β (Fitzgerald) in cell media 4 h before use in flow experiments. HUVEC were also activated with 100 µM histamine (Acros Organics) 2 min before use in flow experiments.

For E-selectin blocking experiments, activated HUVEC were blocked with 20 µg/mL anti-E-selectin (R&D Systems #BBA26) or IgG1 isotype control (BioLegend #400102) for 30 min before use in flow experiments to prevent leukocyte adhesion.

### Platelet adhesion to damaged endothelium under flow

One hour before flow experiments, platelets in whole blood were stained with anti-CD41/61 PE (BioLegend #359806; 1 µg/mL whole blood). To activate platelets and induce expression of P-selectin concurrently with the platelet staining step, 20 µM adenosine diphosphate (ADP, MP Biomedical) was added to aliquots of whole blood 1 h before flow experiments. HUVEC on glass coverslips were manually scored using a scalpel to expose the underlying extracellular matrix^38^. The coverslip was then attached to a parallel plate flow chamber (PPFC, Glycotech) fitted with a silicone gasket (2 cm × 0.25 cm × 127 µm in height). Immediately before blood was perfused over the coverslip, particles were added to whole blood at the specified final concentration in number of particles per mL of whole blood. A syringe was attached to the chamber outlet, and a syringe pump was utilized to control whole blood over the coverslip, with the direction of blood flow perpendicular to the scalpel scores. The wall shear rate was held constant at 1000 s^−1,30^. After 5 min of laminar blood flow, PBS buffer with calcium and magnesium and 1% BSA (pH 7.4) was perfused over the coverslip for 2 min to flush the chamber. After rinsing, a digital camera was utilized to take 10 images of endothelium-bound leukocytes and 10 fluorescent images of bound platelets utilizing a TRITC filter.

### Intravital fluorescence microscopy of inflamed mouse mesentery

Female C57BL/6 mice (aged 3-4 weeks) were used in all assays. Male mice were not included due to the lethal inflammation experienced with IP LPS administration. Mice were anesthetized using isoflurane and given a retro-orbital injection of anti-Ly6G Brilliant Violet 421 (BioLegend #127628; 1.6 µg/mouse), anti-GP1b DyLight 649 (Emfret Analytics X649; 1.8 µg/mouse), and a ~10 mg/kg 2 µm PS particle dosage (~4.5 × 10^7^ particles) for mice receiving a ‘preventative’ particle treatment. Particles were conjugated with anti-E-selectin and anti-ICAM-1 (‘targeted,’ T) or anti-IgG2b (‘untargeted,’ UT) as described above. A separate experimental group was dosed with a 10 mg/kg UT ‘prevention’ of 500 nm PS particles instead (~2.9 × 10^9^ particles). The mice then received an intraperitoneal injection of 5 mg/kg lipopolysaccharide (LPS from *E. coli* O111:B4; Sigma Aldrich) in 100 µL PBS to induce systemic inflammation^49,50^. Mice receiving an ‘intervention’ particle treatment were briefly anesthetized 2 h after LPS IP injection with isoflurane and received 10 mg/kg 2 µm PS via retro-orbital injection. For certain experiments, mice received a 10 mg/kg ‘intervention’ particle treatment of unconjugated (UC) 2 µm PS particles or 2 µm Poly-A particles. Some control mice received an ‘intervention’ of ‘free aspirin’; these mice received a dosage of the total amount of salicylic acid contained in the entire dosage of Poly-A particles suspended in 10% dimethyl sulfoxide (DMSO in PBS) due to low solubility in PBS alone. Separate control mice received an ‘intervention’ of 10% DMSO in PBS (vehicle control). The retro-orbital injection of antibodies for mice receiving Poly-A particles and some control groups consisted of anti-Ly6G Brilliant Violet 421 (Biolegend #127628; 1.6 µg/mouse) and anti-GP1b DyLight 488 (Emfret Analytics X488; 1.8 µg/mouse) as Poly-A particles were fluorescently labeled with Cy5.5.

Three hours after IP LPS injection, mice were anesthetized with a mixture of ketamine and xylazine (~200–250 µL) and mesentery visualized^37,85^. After anesthetization, a midline incision was made, and mesentery exteriorized. Mice were placed on a custom-made microscopic stage with exposed mesentery on a glass coverslip. vessels were visualized using a 25x oil objective on an inverted fluorescence microscope (Zeiss Axio Observer Z1Marianas) using Slidebook 6 software. Vessels ~150 µm in width were imaged every 100 milliseconds using both brightfield and fluorescent microscopy using DAPI, GFP, and Cy5 fluorescent channels to image Ly6G^+^ neutrophils, particles, and GP1b^+^ platelets, respectively. Poly-A and some control mice were instead imaged using DAPI, GFP, and Cy5 fluorescent channels to image Ly6G^+^ neutrophils, GP1b^+^ platelets, and particles, respectively. Each vessel was imaged for 60 frames. 2–4 independent vessels were imaged per mouse.

File names were blinded, and image analysis was performed using ImageJ and Slidebook 6. The number of adherent particles, neutrophils, and platelets were recorded and considered firmly adherent if they did not move over the course of the video. Non-adherent neutrophils passing through the frame were counted. The number of platelets associated with neutrophils was counted; platelets and neutrophils were considered associated if the platelet and neutrophil maintained contact for the entire video. The size of the vessel was calculated using ImageJ, and the number of adherent particles and cells was scaled per mm^2^ of the vessel. Only blood cells and particles within the blood vessel were counted and analyzed.

### Particle biodistribution in mice

1.75 µm PS particles (Polysciences) were conjugated with avidin, as described in above paragraphs. 1.75 µm particles were utilized as they are excited at 641 nm and emit fluorescence at 662 nm, allowing them to be imaged using near-infrared scanners for the purpose of a biodistribution study. The particles were then conjugated with 30,000 sites/µm^2^ anti-IgG2b (untargeted) or 30,000 sites/µm^2^ anti-E-selectin and 20,000 sites/µm^2^ anti-ICAM-1 (targeted) using antibody concentrations described in above paragraphs. Anti-E-selectin and anti-ICAM-1 site densities were quantified via staining with 12 µg/mL anti-rat IgG2a-phycoerthrin (anti-E-selectin; Biolegend #407508) and 12 µg/mL anti-rat IgG2b-phycoerythrin (anti-ICAM-1; Biolegend #408214), as described in above paragraphs. Mice were administered a 5 mg/kg IP injection of LPS. Mice were either kept as untreated controls or given one of the following ~10 mg/kg 1.75 µm PS (~6.8 × 10^7^ particles) particle treatments: UT prevention given at the same time as LPS installation; UT intervention given 2 h after LPS installation; or T intervention given 2 h after LPS installation. 3 h after LPS installation, mice were euthanized. Whole organs (lungs, liver, heart, kidneys, and spleen) and blood were harvested. Whole organs were stored in PBS until scanned. Organs and blood were scanned using an Odyssey CLx Infrared Imaging System (LI-COR) using the 700 nm channel with 169 µm resolution. To calculate the total fluorescence for each organ, a region of interest (ROI) was drawn around each organ using Image Studio Software (LI-COR). Untreated (‘blank’) organs were measured as background fluorescence and subtracted from each particle-treated organ. The percent fluorescence recovered from each organ was found by calculating the mean fluorescent signal of each specific organ divided by the total mean fluorescent signal of all organs for one mouse.

### Neutrophil depletion in mice

For select groups of mice, neutrophils were depleted before LPS installation and intravital microscopy. Each mouse’s baseline complete blood count was measured using saphenous vein blood draws collected into EDTA-coated tubes and measured using a Hemavet Analyzer (Drew Scientific). Then, each mouse was given an IP injection of 500 µg anti-Ly6G depletion antibody (BioLegend #127649). After one day, the complete blood count for each mouse was again measured. Mice were utilized if neutrophil counts were depleted at or below the minimum normal range. Depletion mice received an additional 200 µg anti-Ly6G, administered via retro-orbital injection at the same time as labeling antibodies. A subset of these mice also received a prevention UT treatment of 2 µm PS particles. Like the other experimental groups, systemic inflammation was induced via IP injection of LPS 3 h before intravital microscopy. Neutrophil depletion was again confirmed by measuring the complete blood count of mice at the time of euthanasia via cardiac puncture blood draw.

### Test of Poly-A particle long-term impact on circulating white blood cell distribution and aspartate aminotransferase (AST)

Select groups of mice received a ‘long-term’ dosage of Poly-A. Mice received a daily tail vein injection of 2 × 10^8^ (~1 µm) Poly-A particles in 100 µL sterile phosphate-buffered saline (PBS). Control mice received either an equivalent amount of PBS (‘saline’) alone or ‘free aspirin’—salicylic acid. Mice were weighed and underwent health scoring daily before euthanasia on the fifth day. At the time of euthanasia, a cardiac puncture blood draw was administered, and the liver was harvested. Blood was immediately placed on ice, blocked with 1 µL FC-receptor block (BioLegend #101320) for 10 min. Samples were then stained with 0.1 µg/mL CD45-BV711 (Biolegend #103147), 0.25 µg/mL CD11b-FITC (Biolegend #101206), and 0.1 µg/mL Ly6G-APC (Biolegend #127614) and lyse/fixed (eBioscience) before using an Attune flow cytometer to analyze circulating leukocyte populations. The liver was added to RPMI media with collagenase and DNAse and dissociated using a gentleMACS Dissociator (Miltenyi Biotec). The dissociated liver was strained, centrifuged, and the supernatant was utilized in an aspartate aminotransferase (AST) activity assay (Sigma Aldrich).

### Salicylic acid measurements from mouse plasma

For select groups of mice receiving Poly-A particles or free aspirin, a cardiac puncture blood draw was administered at the time of euthanasia. Plasma was obtained via centrifugation for 15 min at 2000*g*. Salicylic acid was measured in plasma via liquid chromatography-tandem mass spectrometry (LC-MS/MS). This work was done by the Pharmacokinetics Core (PK Core) at the University of Michigan.

### Statistics

For in vitro flow experiments involving HUVEC, 10 non-fluorescent images of bound leukocytes and 10 fluorescent images of bound platelets were taken for each independent experiment with *n* = 5 independent donors for each treatment data point presented. ImageJ was utilized to determine the % area covered of the endothelium by platelets. Each donor treatment was scaled by 1-2 replicates of the donor’s control, or untreated sample, to yield a fold change in platelet or leukocyte adhesion. The background fluorescence for each image was subtracted, and a threshold was set. ImageJ calculated the surface area coverage of each image using the set threshold. ImageJ was also utilized to count adherent leukocytes manually. For in vivo experiments visualizing mouse mesentery vessels, 2–4 independent vessels were imaged per mouse with *n* = 5 mice for each experimental group. For biodistribution studies, *n* = 5 mice were utilized in each experimental group. For salicylic acid plasma measurements, n ≥ 3 independent plasma samples were utilized. For long-term Poly-A circulating leukocyte population distributions and AST activity, n = 3 independent samples were utilized. Data are plotted with standard error bars. Two-tailed unpaired Student’s *t* test or one-, and two-way ANOVA in GraphPad Prism software were used to analyze statistical differences between samples. Asterisks or pound signs indicate *p* values of *<0.05, **<0.01, ***<0.001, and ****<0.0001. A lack of asterisks or pound signs indicate a lack of significance.

### Reporting summary

Further information on research design is available in the Nature Portfolio Reporting Summary linked to this article.

## Supplementary information

Supplementary Information

Description of additional supplementary files

Supplementary Movie 1

Supplementary Movie 2

Supplementary Movie 3

Supplemental Movie 4

Supplemental Movie 5

Supplemental Movie 6

Supplemental Movie 7

Supplemental Movie 8

Supplemental Movie 9

Reporting Summary

## Data Availability

The datasets generated during and/or analyzed during the current study are provided in the Source Data file available at 10.6084/m9.figshare.22583842. All other relevant data supporting the key findings of this study are available within the article and its Supplementary Information files or from the corresponding author upon reasonable request.

## Source data

Source data
