# Supplementary material for: Cargo-free particles divert neutrophil-platelet aggregates to reduce thromboinflammation
Source: Nat Commun. 2023 Apr 28;14:2462. doi: 10.1038/s41467-023-37990-z (PMC10144907; doi:10.1038/s41467-023-37990-z)
Supplement: Supplementary file 1 — Supplementary Information [file 41467_2023_37990_MOESM1_ESM.pdf]

Cargo-Free Particles Divert Neutrophil-Platelet Aggregates to Reduce Thromboinflammation

Alison L. Banka,[1] M. Valentina Guevara,[1] Emma R. Brannon,[1] Nhien Q. Nguyen,[2] Shuang Song,[2] Gillian Cady,[3] David Pinsky,[3] Kathryn E. Uhrich,[2] Reheman Adili,[4] Michael Holinstat,[3,4] and Omolola Eniola-Adefeso[1,5,6]

1. Department of Chemical Engineering, University of Michigan, Ann Arbor, MI 48109
2. Department of Chemistry, University of California Riverside, Riverside, CA 92521
3. Division of Cardiovascular Medicine, Samuel and Jean Frankel Cardiovascular Center, University of Michigan, Ann Arbor, MI 48109
4. Department of Pharmacology, University of Michigan, Ann Arbor, MI 48109
5. Department of Biomedical Engineering, University of Michigan, Ann Arbor, MI 48109.
6. Macromolecular Science and Engineering Program, University of Michigan, Ann Arbor, MI 48109.

Correspondence should be addressed to O. E-A. (email: lolaa@umich.edu).

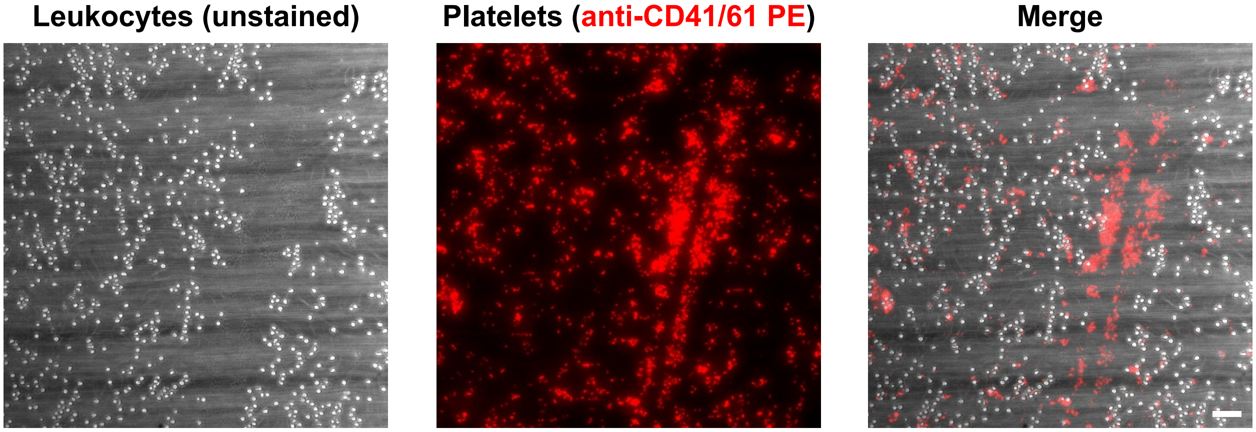

Supplementary Figure 1: Activation of endothelium and platelets leads to adhesion of platelet-leukocyte aggregates. Representative images of leukocytes (via brightfield microscopy) and platelets (stained with anti-CD41/61 PE, red, via fluorescent microscopy) adherent to a HUVEC monolayer after 5 minutes of laminar blood flow at $1000s^{-1}$ in whole blood. HUVEC were activated for 4 hours with IL-1β and platelets were activated for 1 hour with ADP prior to blood flow, scale bar 100 µm.

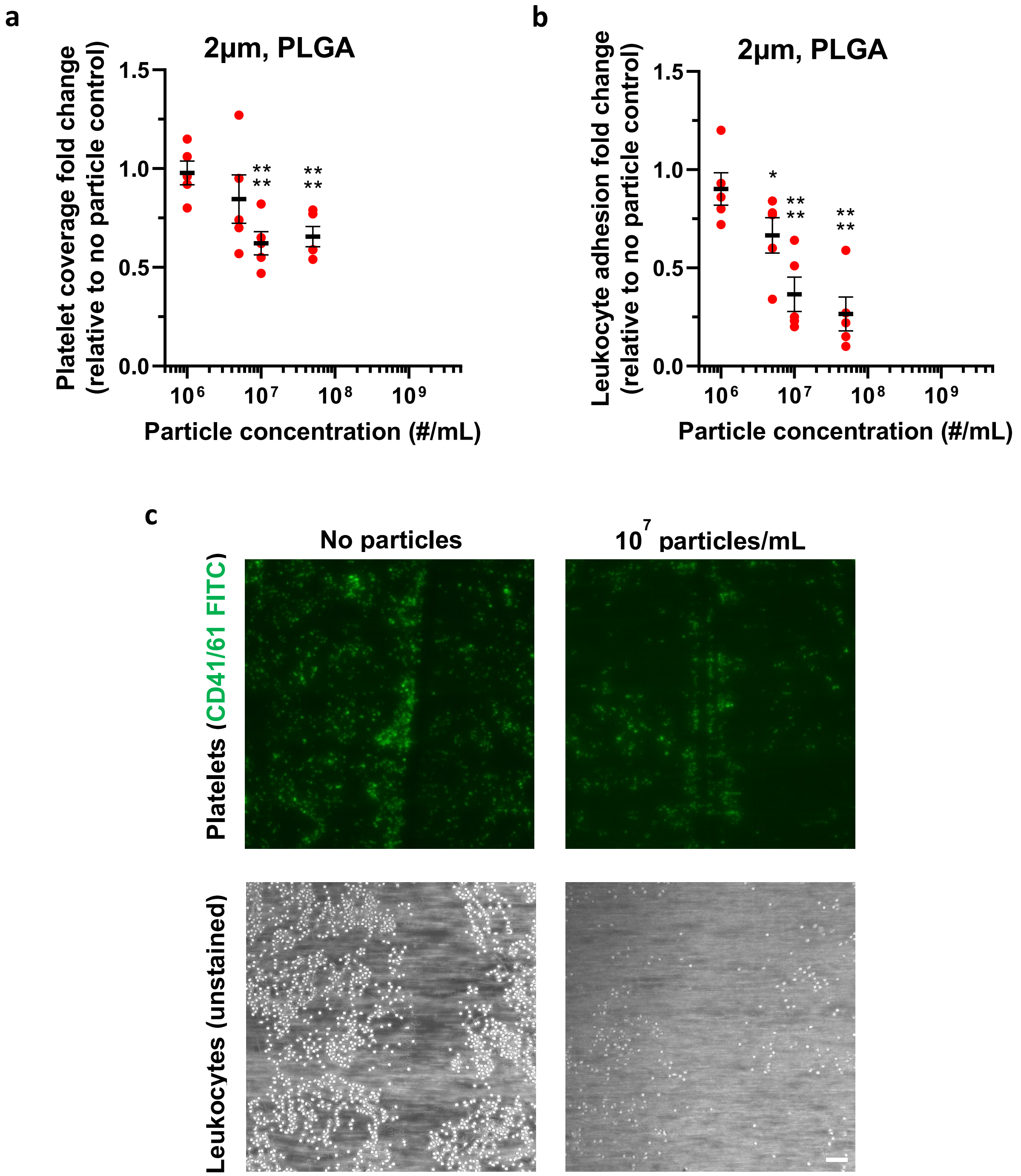

Supplementary Figure 2: Micron-sized, biodegradable PLGA particles decrease platelet-leukocyte aggregate adhesion to an inflamed endothelium. Impact of ~2 µm IgG-conjugated PLGA particles on platelet adhesion (A) and leukocyte adhesion (B) to an inflamed, damaged endothelium in whole blood flow in comparison to non-particle controls, (C) Representative images of adherent platelets (green) and leukocytes (unstained) to inflamed, damaged HUVEC monolayer in the absence (left) or presence (right) of $1*10^7$/mL ~2 µm IgG-conjugated PLGA particles. N = 5 independent donors were utilized for each particle treatment, with 2 replicates per donor utilized for each (non-treatment) control. Statistical analyses were performed using two-way ANOVA with Sidak's multiple comparisons test. (*) indicates $p<0.05$, and (****) indicates $p<0.0001$ in comparison to no particle controls. Lack of symbols indicates no statistical

significance. Circles represent individual data points, horizontal bar represents the average of individual data points, error bars represent standard error, and scale bar = 100 μm. Source data and specific p-values are provided as a Source Data file.

a

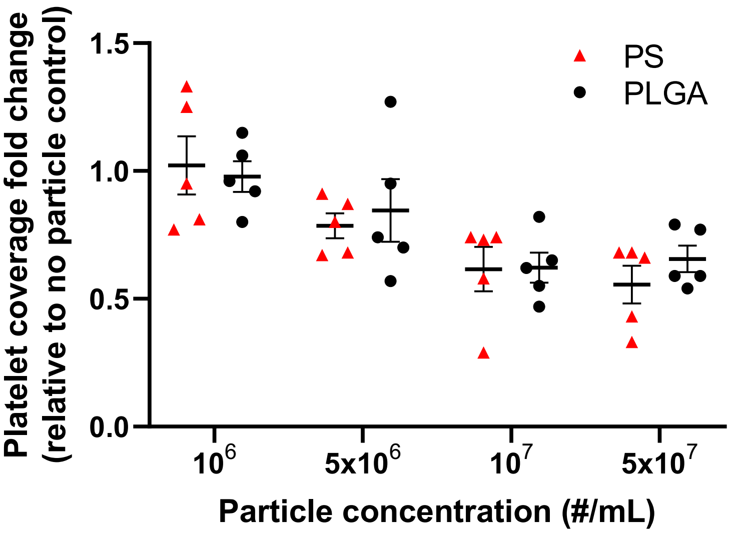

b

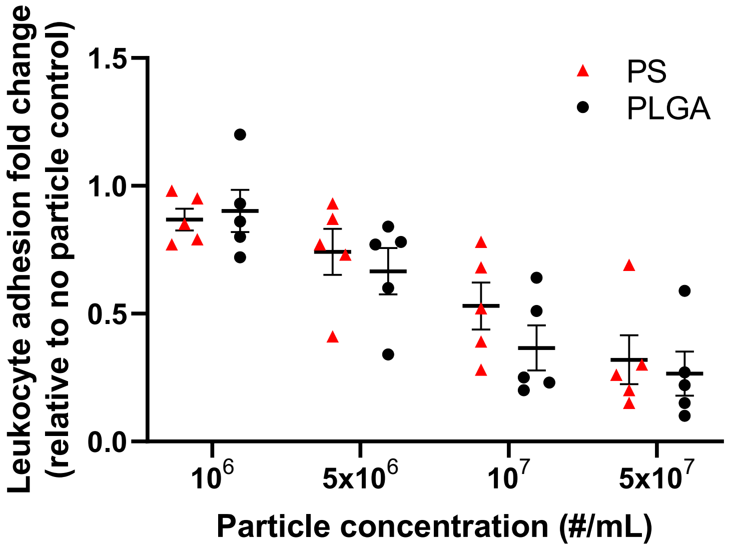

Supplementary Figure 3: Micron-sized PS and PLGA particles lead to a similar decrease in platelet and leukocyte adhesion *in vitro*. Impact of 2 µm IgG-conjugated model PS (filled) and PLGA (patterned) particles on platelet adhesion (A) and leukocyte adhesion (B) to an inflamed, damaged endothelium in whole blood flow in comparison to non-particle controls. N = 5 independent donors were utilized for each particle treatment with 2 replicates per donor utilized for each (non-treatment) control. Statistical analyses were performed using a two-way ANOVA with Sidak's multiple comparisons test. Lack of symbols indicates no statistical significance. Circles and triangles represent individual data points, horizontal bar

represents the average of individual data points, and error bars represent standard error. Source data and specific p-values are provided as a Source Data file.

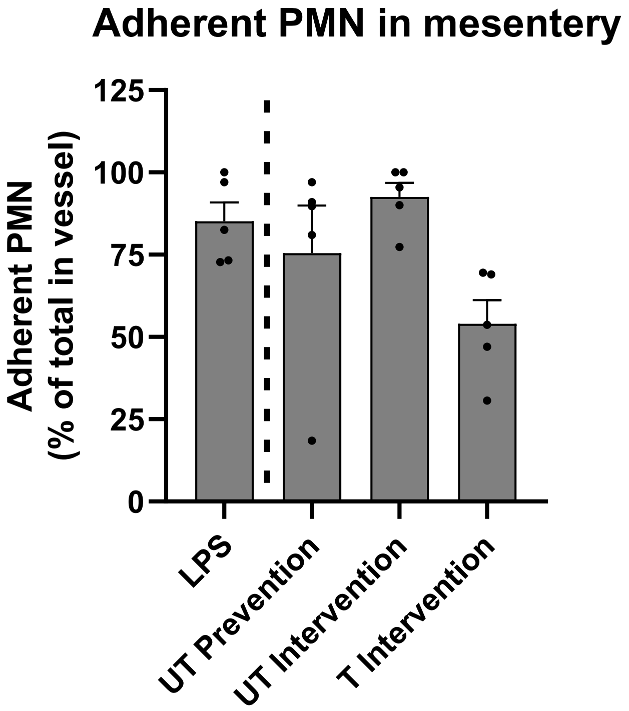

Supplementary Figure 4: PS particle treatment does not change the proportion of firmly adherent neutrophils in inflamed mouse mesentery. The percent of all PMN (adherent, rolling, and in blood flow) that are firmly adherent to the mesentery wall over a 60-frame video. Videos were taken 3 hours after IP injection of LPS. Experimental groups consist of N=5 mice per group with 2-4 independent vessels imaged per mouse. Circles represent individual data points, bar graph represents the average of individual data points, and error bars represent standard error. Source data and specific p-values are provided as a Source Data file.

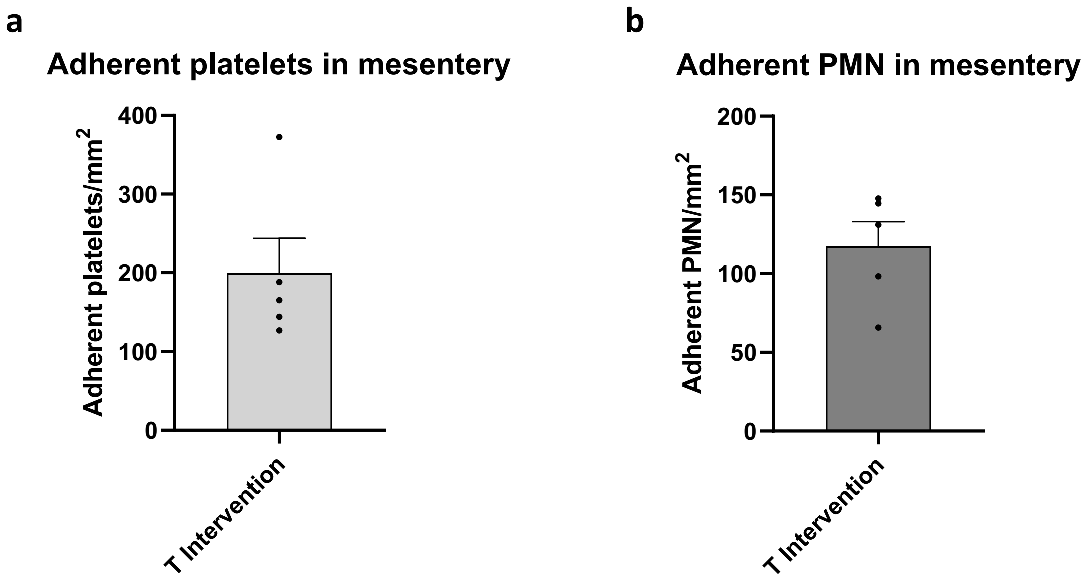

Supplementary Figure 5: Platelet and neutrophil adhesion in mouse mesentery after injection of targeted particles (T). Quantified results of (a) platelet adhesion, and (b) neutrophil adhesion (PMN) in the mouse mesentery 3 hours after IP injection of LPS and 1 hour after the injection of intervention T particles, scaled by the surface area of the blood vessel. Experimental groups consist of N=5 mice per group with 2-4 independent vessels imaged per mouse. Circles represent individual data points, bar graph represents the average of individual data points, and error bars represent standard error. Source data and specific p-values are provided as a Source Data file.

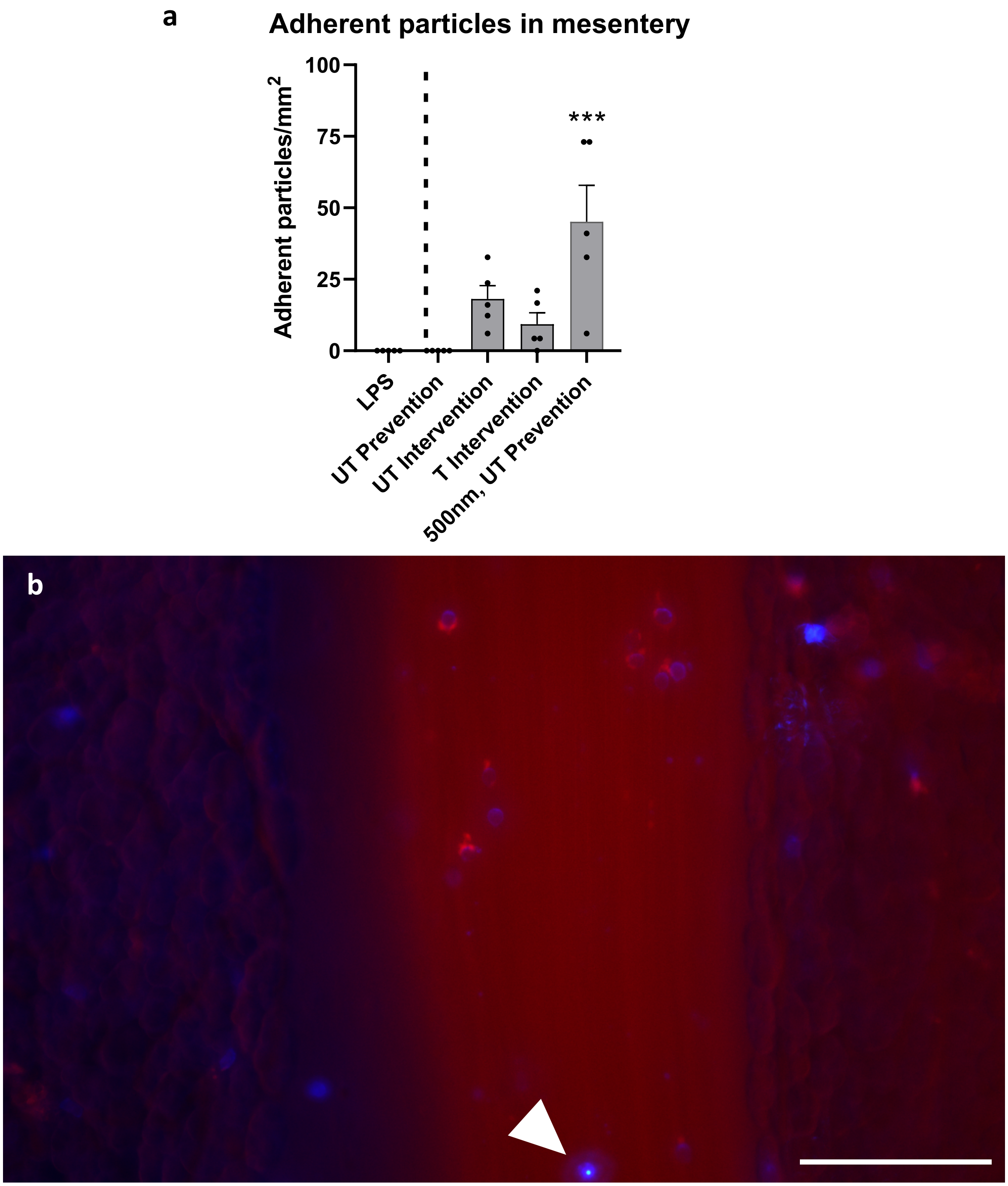

Supplementary Figure 6: Minimal particles bound to inflamed mouse mesentery wall. (A) Quantified results of particle adhesion in mouse mesentery 3 hours after IP injection of LPS, scaled by the surface area of the blood vessel. (B) Representative merged image of a mouse mesenteric blood vessel after 3-hour IP LPS injection and UT intervention 2 µm PS particle injection with channels fluorescent Brilliant Violet 421 Ly6G$^+$ neutrophils (blue), FITC polystyrene particles (green), anti-GP1b DyLight 649 platelets (red), scale bar 100 µm. White arrow highlights a green (FITC) particle associated with bound neutrophil. Experimental groups consist of N=5 mice per group with 2-4 independent vessels imaged per mouse. Statistical analyses were performed using a one-way ANOVA with Dunnett's multiple comparison test. (***) indicates p <

0.001 in comparison to LPS-only controls. Lack of symbols indicates no statistical significance. Circles represent individual data points, bar graph represents the average of individual data points, and error bars represent standard error. Source data and specific p-values are provided as a Source Data file.

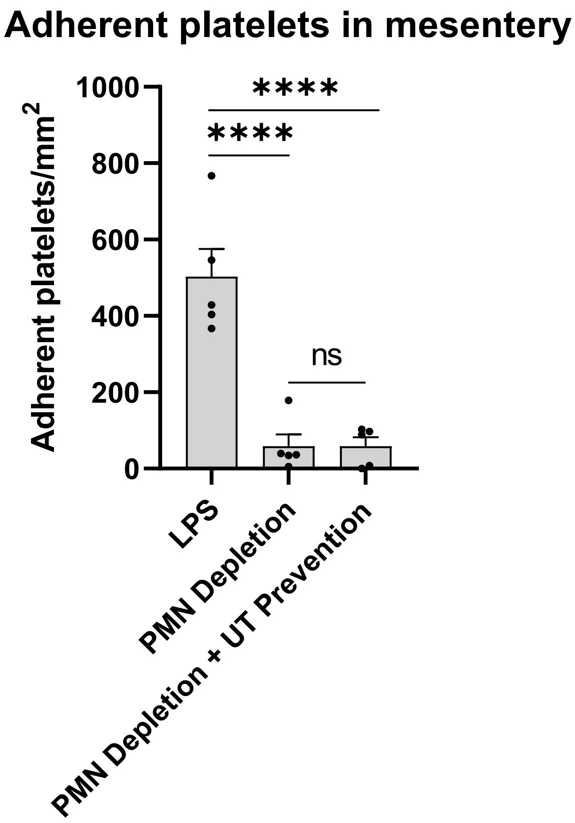

Supplementary Figure 7: Depletion of neutrophils reduces platelet adhesion and impact of particles on platelet adhesion in inflamed mouse mesentery *in vivo*. Quantified results of platelet adhesion in the mouse mesentery 3 hours after IP injection of LPS and after depletion of neutrophils, scaled by the surface area of the blood vessel. Experimental groups consist of N=5 mice per group with 2-4 independent vessels imaged per mouse. Statistical analyses were performed using a one-way ANOVA with Tukey's multiple comparisons test. (****) indicates $p<0.0001$ in comparison to LPS-only controls. 'NS' indicates no statistical significance. Circles represent individual data points, bar graph represents the average of individual data points, and error bars represent standard error. Source data and specific p-values are provided as a Source Data file.

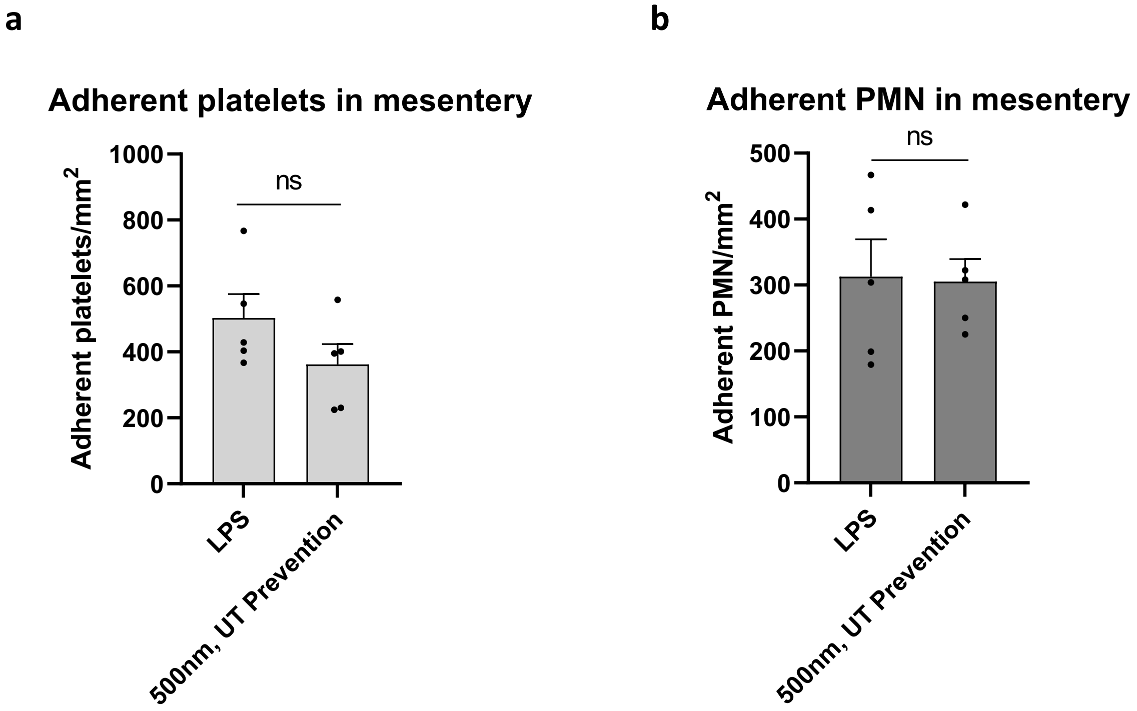

Supplementary Figure 8: Untargeted, nano-sized particles do not reduce platelet or neutrophil accumulation in inflamed mouse mesentery. Quantified results of (A) platelet adhesion, and (B) neutrophil (PMN) adhesion in mouse mesentery 3 hours after IP injection of LPS, scaled by the surface area of the blood vessel. Experimental groups consist of N=5 mice per group with 2-4 independent vessels imaged per mouse. Statistical analyses were performed using an unpaired Student's t-test. 'NS' indicates no statistical significance. Circles represent individual data points, bar graph represents the average of individual data points, and error bars represent standard error. Source data and specific p-values are provided as a Source Data file.

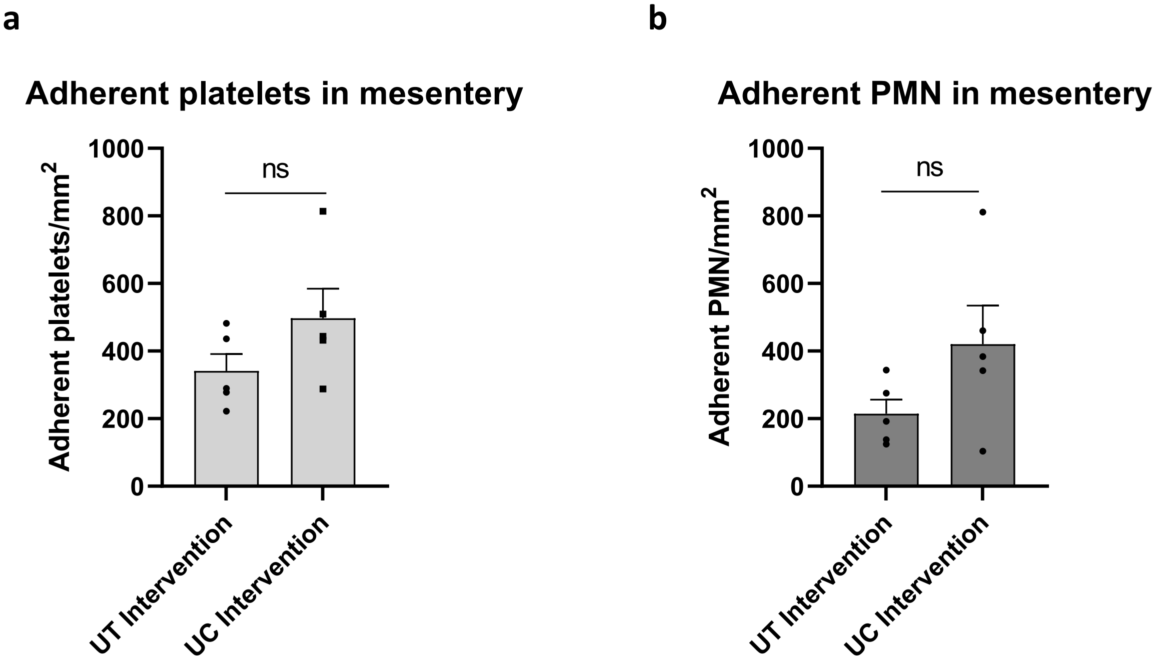

Supplementary Figure 9: UT Intervention PS particles yield same impact on platelet and neutrophil accumulation in mouse mesentery as UC Intervention PS particles. Quantified results of (A) platelet adhesion, and (B) neutrophil (PMN) adhesion in mouse mesentery 3 hours after IP injection of LPS, scaled by the surface area of the blood vessel. PS particles were either conjugated with avidin and anti-IgG ('UT,' left) or not conjugated ('UC,' right). Experimental groups consist of N=5 mice per group with 2-4 independent vessels imaged per mouse. Statistical analyses were performed using an unpaired Student's t-test. 'NS' indicates no statistical significance. Circles represent individual data points, bar graph represents the average of individual data points, and error bars represent standard error. Source data and specific p-values are provided as a Source Data file.

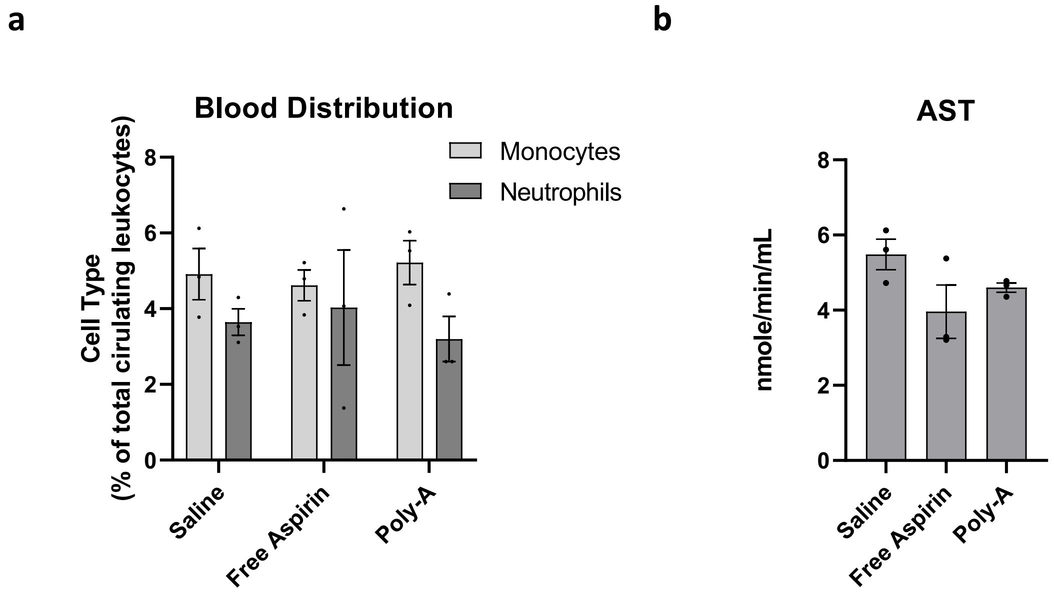

Supplementary Figure 10: Repeated administration of Poly-A particles does not impact circulating blood types or liver function in mice. Quantified results of (A) the distribution of circulating leukocytes, and (B) liver aspartate aminotransferase (AST) activity in mice receiving repeated doses of Poly-A particles, saline, or free aspirin. Poly-A mice received $2*10^8$ particles daily over the course of five days with analysis after euthanasia on the final day. Experimental groups consist of N=3 mice per group. Statistical analyses were performed using a two-way ANOVA with Tukey's multiple comparisons test (A) and a one-way ANOVA with Tukey's multiple comparisons test (B). Lack of symbols indicates no statistical significance. Circles represent individual data points, bar graph represents the average of individual data points, and error bars represent standard error. Source data and specific p-values are provided as a Source Data file.

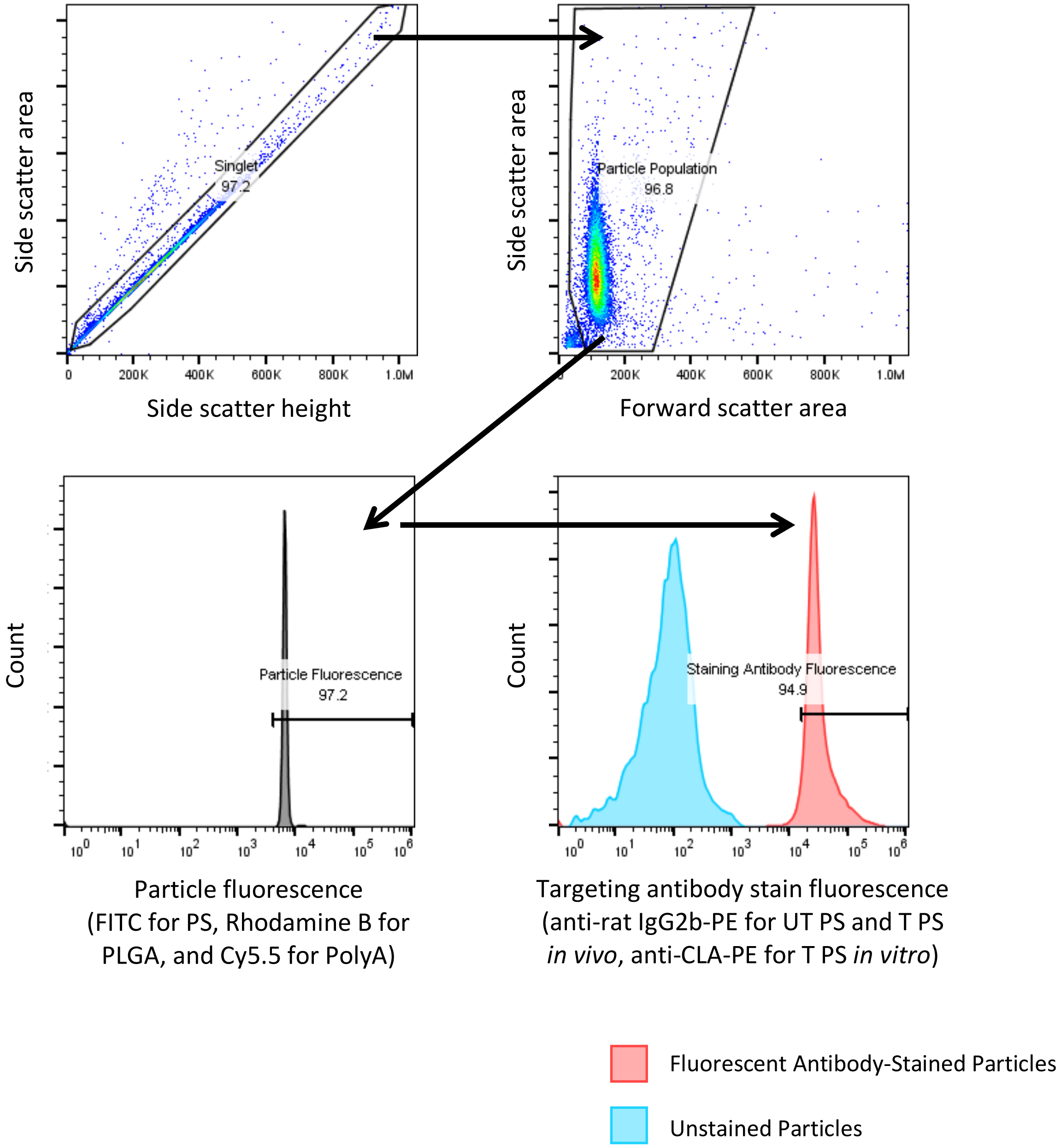

Supplementary Figure 11: Representative flow cytometry gating strategy for conjugated particles. Polystyrene (PS), poly-lactic-co-glycolic acid (PLGA), or salicylate-based poly(anhydride-ester) (Poly-A) particles were conjugated with anti-IgG2b (‘untargeted,’ UT) or targeting ligands (‘targeted,’ T) and stained with a fluorescent antibody. Stained particles were analyzed via flow cytometry for their median fluorescence by first gating single particles, then gating the particle population, gating for the fluorescence of the particle, then finally the fluorescence of the antibody stain. The fluorescent marker for PS particles was fluorescein isothiocyanate (FITC), PLGA particles was Rhodamine B, and Poly-A particles was Cyanine-5.5 (Cy5.5).

Supplementary Table 1: Sizes and surface charges of ~2 µm particles used *in vitro* and *in vivo*.

Particle Type	Size (µm)	Zeta Potential (mV)
PS	2.08 ± 0.10	-43.0
PLGA	1.83 ± 0.71	-18.8
Poly-A	2.40 ± 0.47	-31.8
