# Supplementary material for: Cargo-free particles divert neutrophil-platelet aggregates to reduce thromboinflammation
Source: Nat Commun. 2023 Apr 28;14:2462. doi: 10.1038/s41467-023-37990-z (PMC10144907; doi:10.1038/s41467-023-37990-z)
Supplement: Supplementary file 2 — Description of additional supplementary files [file 41467_2023_37990_MOESM2_ESM.pdf]

Description of additional supplementary files

Supplementary Movie 1: Video of neutrophil and platelet adhesion to the mesentery of an LPS-only treated mouse. Neutrophils are labelled with Brilliant Violet 421 Ly6G$^+$ (blue) and platelets are labelled with anti-GP1b DyLight 649 (red).

Supplementary Movie 2: Video showing no neutrophil and minimal platelet adhesion to the mesentery for a non-LPS control mouse. Neutrophils are labelled with Brilliant Violet 421 Ly6G$^+$ (blue) and platelets are labelled with anti-GP1b DyLight 649 (red).

Supplementary Movie 3: Video of neutrophil and platelet adhesion to the mesentery in an LPS-treated mouse receiving a UT prevention particle treatment. Neutrophils are labelled with Brilliant Violet 421 Ly6G$^+$ (blue), platelets are labelled with anti-GP1b DyLight 649 (red), and PS particles are labelled with FITC (green).

Supplementary Movie 4: Video of neutrophil and platelet adhesion to the mesentery in an LPS-treated mouse receiving a UT intervention particle treatment. Neutrophils are labelled with Brilliant Violet 421 Ly6G$^+$ (blue), platelets are labelled with anti-GP1b DyLight 649 (red), and PS particles are labelled with FITC (green).

Supplementary Movie 5: Video of neutrophil and platelet adhesion to the mesentery in an LPS-treated mouse receiving a T intervention particle treatment. Neutrophils are labelled with Brilliant Violet 421 Ly6G$^+$ (blue), platelets are labelled with anti-GP1b DyLight 649 (red), and PS particles are labelled with FITC (green).

Supplementary Movie 6: Video demonstrating minimal neutrophil and platelet adhesion to the mesentery in a neutrophil-depleted, LPS-treated mouse. Neutrophils are labelled with Brilliant Violet 421 Ly6G$^+$ (blue) and platelets are labelled with anti-GP1b DyLight 649 (red).

Supplementary Movie 7: Video demonstrating minimal neutrophil and platelet adhesion to the mesentery in a neutrophil-depleted, LPS-treated mouse receiving a UT prevention particle treatment. Neutrophils are labelled with Brilliant Violet 421 Ly6G$^+$ (blue), platelets are labelled with anti-GP1b DyLight 649 (red), and PS particles are labelled with FITC (green).

Supplementary Movie 8: Video of neutrophil and platelet adhesion to the mesentery in an LPS-treated mouse receiving a UT prevention nanoparticle treatment. Neutrophils are labelled with Brilliant Violet 421 Ly6G$^+$ (blue), platelets are labelled with anti-GP1b DyLight 649 (red), and PS particles are labelled with FITC (green).

Supplementary Movie 9: Video of neutrophil and platelet adhesion to the mesentery in an LPS-treated mouse receiving a UC Poly-A intervention particle treatment. Neutrophils are labelled with Brilliant Violet 421 Ly6G$^+$ (blue), platelets are labelled with anti-GP1b DyLight 488 (green), and Poly-A particles are labelled with Cy5.5 (red).
