# Supplementary material for: Cargo-free particles divert neutrophil-platelet aggregates to reduce thromboinflammation
Source: Nat Commun. 2023 Apr 28;14:2462. doi: 10.1038/s41467-023-37990-z (PMC10144907; doi:10.1038/s41467-023-37990-z)
Supplement: Supplementary file 12 — Reporting Summary [file 41467_2023_37990_MOESM12_ESM.pdf]

Corresponding author(s): Eniola-Adefeso

Last updated by author(s): Apr 2, 2023

Reporting Summary

Nature Portfolio wishes to improve the reproducibility of the work that we publish. This form provides structure for consistency and transparency in reporting. For further information on Nature Portfolio policies, see our Editorial Policies and the Editorial Policy Checklist.

Statistics

For all statistical analyses, confirm that the following items are present in the figure legend, table legend, main text, or Methods section.

n/a	Confirmed	
☐	☒	The exact sample size (*n*) for each experimental group/condition, given as a discrete number and unit of measurement
☐	☒	A statement on whether measurements were taken from distinct samples or whether the same sample was measured repeatedly
☐	☒	The statistical test(s) used AND whether they are one- or two-sided<br>*Only common tests should be described solely by name; describe more complex techniques in the Methods section.*
☒	☐	A description of all covariates tested
☐	☒	A description of any assumptions or corrections, such as tests of normality and adjustment for multiple comparisons
☐	☒	A full description of the statistical parameters including central tendency (e.g. means) or other basic estimates (e.g. regression coefficient) AND variation (e.g. standard deviation) or associated estimates of uncertainty (e.g. confidence intervals)
☐	☒	For null hypothesis testing, the test statistic (e.g. *F*, *t*, *r*) with confidence intervals, effect sizes, degrees of freedom and *P* value noted<br>*Give P values as exact values whenever suitable.*
☒	☐	For Bayesian analysis, information on the choice of priors and Markov chain Monte Carlo settings
☒	☐	For hierarchical and complex designs, identification of the appropriate level for tests and full reporting of outcomes
☒	☐	Estimates of effect sizes (e.g. Cohen's *d*, Pearson's *r*), indicating how they were calculated

Our web collection on statistics for biologists contains articles on many of the points above.

Software and code

Policy information about availability of computer code

Data collection	Data collected via microscopy software (Slidebook 6, NIS-Elements)
Data analysis	GraphPad Prism 9; ImageJ 1.53e

For manuscripts utilizing custom algorithms or software that are central to the research but not yet described in published literature, software must be made available to editors and reviewers. We strongly encourage code deposition in a community repository (e.g. GitHub). See the Nature Portfolio guidelines for submitting code & software for further information.

Data

Policy information about availability of data

All manuscripts must include a data availability statement. This statement should provide the following information, where applicable:

- Accession codes, unique identifiers, or web links for publicly available datasets
- A description of any restrictions on data availability
- For clinical datasets or third party data, please ensure that the statement adheres to our policy

The data generated in this study are provided in the Supplementary Information/Source Data file.

Human research participants

Policy information about studies involving human research participants and Sex and Gender in Research.

Reporting on sex and gender	Findings apply to male and female sex. Gender data was not collected.
Population characteristics	Covariate-relevant population characteristics were not collected in this study, as per our IRB which does not allow for collection of this data.
Recruitment	Participants were recruited via flyer and email advertisements to nearby university community, skewing the population of participants towards a college-educated population.
Ethics oversight	University of Michigan Internal Review Board

Note that full information on the approval of the study protocol must also be provided in the manuscript.

Field-specific reporting

Please select the one below that is the best fit for your research. If you are not sure, read the appropriate sections before making your selection.

☒ Life sciences ☐ Behavioural & social sciences ☐ Ecological, evolutionary & environmental sciences

For a reference copy of the document with all sections, see nature.com/documents/nr-reporting-summary-flat.pdf

Life sciences study design

All studies must disclose on these points even when the disclosure is negative.

Sample size	A sample size of N=5 was chosen for in vitro flow assays and in vivo intravital microscopy. Historically, N=5 has been utilized in our laboratory for both in vitro and in vivo work and provides sufficient samples to note trends, even if there is notable variation between human donors or mice.
Data exclusions	Only human donors were utilized that had substantial eukocyte adhesion to an inflamed endothelium (150 leukocytes per image)-- if leukocyte binding was low, endothelial cells were freshly isolated for use as low adhesion was a sign of aging endothelial cells not fully responding to inflammatory stimuli. Mice were excluded if they did not respond to ketamine/xylazine anesthesia within 30 minutes of administration or if retro-orbital injections were not successful.
Replication	Findings were verified utilizing particles of different material (data within text). Intravital microscopy data was validated in a second set of experiments several months later.
Randomization	Mice were randomly assigned to experimental groups. Human subjects served as their own 'non-particle controls' and were also randomly assigned to different particle groups.
Blinding	Blinding was not done for in vitro experiments as the data analysis method relied upon ImageJ (automated). Animal groups were blinded to the analyzer and were only unblinded when compiling data into experimental groups.

Reporting for specific materials, systems and methods

We require information from authors about some types of materials, experimental systems and methods used in many studies. Here, indicate whether each material, system or method listed is relevant to your study. If you are not sure if a list item applies to your research, read the appropriate section before selecting a response.

Materials & experimental systems

n/a	Involved in the study	
☐	☒	Antibodies
☒	☐	Eukaryotic cell lines
☒	☐	Palaeontology and archaeology
☐	☒	Animals and other organisms
☒	☐	Clinical data
☒	☐	Dual use research of concern

Methods

n/a	Involved in the study	
☒	☐	ChIP-seq
☐	☒	Flow cytometry
☒	☐	MRI-based neuroimaging

Antibodies

Antibodies used	anti-rat IgG2b-phycoerythrin (Biolegend) anti-CLA-PE (Miltenyi Biotec); anti-E-selectin (R&D Sytems); anti-ICAM-1 (Biolegend); biotin rat IgG2b (Biolegend); anti-CD41/61 PE (Biolegend); anti-CD41/61 FITC (Biolegend); anti-Ly6G Brilliant Violet 421 (Biolegend);anti-GP1b DyLight 649 (Emfret Analytics); anti-GP1b DyLight 488 (Emfret Analytics); anti-Ly6G (Biolegend)
Validation	Antibodies were validated by manufacturer before purchase and were utilized in previous peer-reviewed publications as provided by the manufacturer.

Animals and other research organisms

Policy information about studies involving animals; ARRIVE guidelines recommended for reporting animal research, and Sex and Gender in Research

Laboratory animals	C57BL/6 mice, aged 3-4 weeks
Wild animals	Study did not utilize wild animals
Reporting on sex	Female mice were used only due to the difference in magnitude of inflammation seen between male and female mice after administration of IP LPS. Specifically, neutrophils accumulated in the mesentery in male mice at a lower LPS concentration than female mice. Female mice were thus chosen as male mice experienced inflammation in the mesentery to an even higher extent.
Field-collected samples	No samples were collected from field.
Ethics oversight	Animal studies were conducted in accordance with National Institutes of Health guidance. Animal studies were approved by the University of Michigan Institutional Animal Care and Use Committee.

Note that full information on the approval of the study protocol must also be provided in the manuscript.

Flow Cytometry

Plots

Confirm that:

☒ The axis labels state the marker and fluorochrome used (e.g. CD4-FITC).

☒ The axis scales are clearly visible. Include numbers along axes only for bottom left plot of group (a 'group' is an analysis of identical markers).

☒ All plots are contour plots with outliers or pseudocolor plots.

☒ A numerical value for number of cells or percentage (with statistics) is provided.

Methodology

Sample preparation	Flow cytometry was primarily used for analysis of targeting ligands on polymeric particles (not from a biological source)
Instrument	Attune NxT Flow Cytometer
Software	FlowJo 10.6.0
Cell population abundance	At least 30K particles (single particles, population identified by forward and side scatter) were analyzed for each condition
Gating strategy	Single particles were gated, then identified by forward and side scatter, and then identified via particle fluorescent color

☒ Tick this box to confirm that a figure exemplifying the gating strategy is provided in the Supplementary Information.
